# The potential impact of exercise on affect and neuroinflammation in older adults living with fibromyalgia: a scoping review

**DOI:** 10.3389/fnhum.2024.1463935

**Published:** 2025-01-06

**Authors:** Taylor L. Taylor, Fitzgerald Dodds, McKenna Tharpe, Emily L. Zumbro, Michael Hankes, Raymond Jones, Deanna Rumble, Lisa Antoine, Kristen Allen-Watts, Andrew Sims, Reshu Chandra, Burel R. Goodin, Jarred Younger, Thomas W. Buford

**Affiliations:** ^1^Department of Family and Community Medicine, Heersink School of Medicine, University of Alabama at Birmingham, Birmingham, AL, United States; ^2^Department of Physical Therapy, School of Health Professions, University of Alabama at Birmingham, Birmingham, AL, United States; ^3^Department of Medicine, Heersink School of Medicine, University of Alabama at Birmingham, Birmingham, AL, United States; ^4^Department of Psychology and Counseling, University of Central Arkansas, Conway, AR, United States; ^5^Department of Neurology, University of Alabama at Birmingham, Birmingham, AL, United States; ^6^Department of Medicine, University of Mississippi Medical Center, Jackson, MS, United States; ^7^Heersink School of Medicine, University of Alabama at Birmingham, Birmingham, AL, United States; ^8^Department of Anesthesiology, Washington University Pain Center, Washington University, St. Louis, MO, United States; ^9^Department of Psychology, University of Alabama at Birmingham, Birmingham, AL, United States; ^10^Department of Veterans Affairs, Birmingham/Atlanta Geriatric Research, Education, and Clinical Center (GRECC), Birmingham, AL, United States

**Keywords:** exercise, affect, neuroinflammation, older adults, fibromyalgia

## Abstract

**Introduction:**

Fibromyalgia (FM) is a widespread chronic pain condition with prevalence increasing in older adults. Older adults living with FM experience longer pain symptom durations that can negatively impact their quality of life. Affect and neuroinflammation are potential factors that can exacerbate pain symptoms. Exercise is a recommended intervention to manage pain symptoms; however, adherence limitations persist. Drawing on the Biopsychosocial Framework of Chronic Pain, this scoping review explores how exercise impacts factors related to neuroinflammation and affect, and how these factors contribute to exercise adherence in older adults living with FM.

**Methods:**

We conducted a scoping search of articles related to exercise and older adults living with FM published before 2024. The extracted study characteristics include publication type, study design, affect outcomes, neuroinflammation outcomes, exercise type, exercise adherence, and sample demographic information.

**Results:**

We have provided an overview of the relationship between affect and neuroinflammation in studies including older adults living with FM and highlight the impact of exercise on affect and neuroinflammation in older adults living with FM. A conceptual framework is provided illustrating the reciprocal relationship between exercise, affective changes, neuroinflammation, and exercise adherence.

**Discussion:**

Our results suggest that exercise may improve affect, while limited evidence suggests that aerobic and resistance exercise improve neuroinflammation. Finally, implications for importance and future directions in the context of potential biological factors impacted are provided.

## 1 Introduction

Fibromyalgia (FM) is a chronic pain condition estimated to affect approximately 2% of the population, ultimately representing 5 million people worldwide (Wolfe et al., 1995). FM is characterized by widespread pain, sleep problems, fatigue, and emotional distress that can result in enormous individual and financial burdens (Penacoba Puente et al., 2013). Timing of when patients present their symptoms and the number of symptoms presented, and the variability of symptoms presented to care providers can result in delays in diagnosis (Choy et al., 2010). Additionally, the burden associated with FM can have societal implications affecting work productivity and even job loss (Annemans et al., 2009). Diagnosis of FM is particularly common in middle age, with the risk of developing FM increasing as individuals age (Ubago et al., 2008). Previous evidence suggests exercise has physical, emotional, and social benefits for individuals living with FM (Bidonde et al., 2019). However, many people living with FM do not experience these benefits due to poor adherence to exercise programs for reasons related to their FM pain symptoms (Andrade et al., 2020).

Psychological factors are powerful predictors of the experience of pain in persons living with FM (Hassett et al., 2008). Dispositional trait styles, or the magnitude of positive affect (PA) and negative affect (NA), are modeled as either protective or vulnerable factors for affective experiences, including pain (Diatchenko et al., 2006; Gertler et al., 2023; Sambuco et al., 2021). Protective dispositional trait styles are categorized as healthy (high PA/low NA) or low (low PA/low NA), whereas vulnerable dispositional trait styles are categorized as reactive (high PA/high NA) and depressive (low PA/high NA) (Sibille et al., 2012). Vulnerable dispositional traits are related to psychiatric comorbidities and are the predominant trait type in patients living with FM (Hassett et al., 2008). However, they have the potential to be modified over time with PA promoting interventions (Sambuco et al., 2022).

Stressful experiences and certain negative emotions, moods, and traits are linked to higher systemic levels of proinflammatory cytokines (Lu et al., 2013). Additionally, accumulating evidence suggests that common pro-inflammatory cytokines, such as IL-1β, IL-6, and TNF-*α*, participate in the pathogenesis of chronic pain and FM (Menzies and Lyon, 2010; Zhang and An, 2007). Further, pro-inflammatory cytokines are potentially linked to microglia-derived neuroinflammation via the blood brain barrier (Kiguchi et al., 2012). Activation of microglia facilitates subsequent production of similar cytokines, with chronic activation often manifesting as fatigue and negative mood symptoms related to FM (Donnelly et al., 2020; Kiguchi et al., 2012). Recently, neuroinflammation is recognized as a physiological mechanism directly associated with changes in dysregulated affect and chronic pain (Albrecht et al., 2021). For example, increased levels of neuroinflammatory markers, such as microglia, chemokines, and brain metabolites are identified in patients with chronic pain and depression (Jung et al., 2023; Troubat et al., 2021).

Exercise is well-established at regulating affect and promoting anti-inflammatory effects, making it a potential strategy to both improve foundational aspects of dispositional traits and reduce inflammation that contributes to FM pain (Chan et al., 2019; Gleeson et al., 2011). Thus, understanding the role of exercise on such mechanisms in patients with chronic inflammatory and musculoskeletal diseases, such as FM is necessary to promote chronic pain management. Regular exercise induces multiple physiological adaptations that can mitigate the pathophysiological mechanisms contributing to enhanced pain dysregulation, including eliciting anti-inflammatory effects in patients living with FM (Ortega et al., 2009). However, recent data suggests an acute bout of exercise can exacerbate pain and fatigue in those living with FM (Naugle et al., 2012). This may lead to a fear avoidance of exercise, creating an emotional barrier that can also critically influence exercise adherence (Vader et al., 2021).

While there is no cure for FM, existing treatments focus on symptom relief. Treatments include cognitive behavioral therapy, medications (i.e., antidepressants, anti-seizure, and analgesics), and complementary and integrative medical therapies (i.e., acupuncture, massage, and hypnosis) (Ram et al., 2023). Exercise is an advantageous cost-effective strategy that, unlike other treatments, provides beneficial effects to multiple FM symptoms at the biological, psychological, and social levels (Bidonde et al., 2019). Exercise has a beneficial affective and inflammatory effect but linking it to neuroinflammation adds a new dimension that may indicate novel therapeutic strategies. However, the relationship between exercise, neuroinflammation, and affect for pain in older adults living with FM, with inflammation serving as a potential mediator are left unexplored. We are addressing gaps not previously addressed in the literature specific to older adults living with FM by reviewing exercise-related changes in affect, neuroinflammation, and inflammation. Additionally, we are systematically examining the barriers and facilitators impacting exercise adherence in this population. To achieve this premise, first, an overview of the relation between neuroinflammation and inflammation in FM is presented. Second, the relationship between affect, neuroinflammation, and inflammation is presented within the context of FM. Third, exercise effects are provided to highlight the impact of exercise on affect and neuroinflammation in individuals living with FM. A conceptual framework is provided to depict how exercise alters these factors with potential implications for FM. Finally, significance and future directions for testing the outlined conceptual framework are provided.

## 2 Materials and methods

### 2.1 Protocol

This scoping review was developed in accordance with the Preferred Reporting Items for Systematic Reviews and Meta-Analyses (PRISMA) extension for scoping reviews (PRISMA-ScR) aimed to address the influence of exercise on affect and neuroinflammation in older adults living with FM (Arksey and O’Malley, 2005).

### 2.2 Eligibility criteria

**Figure 1:**
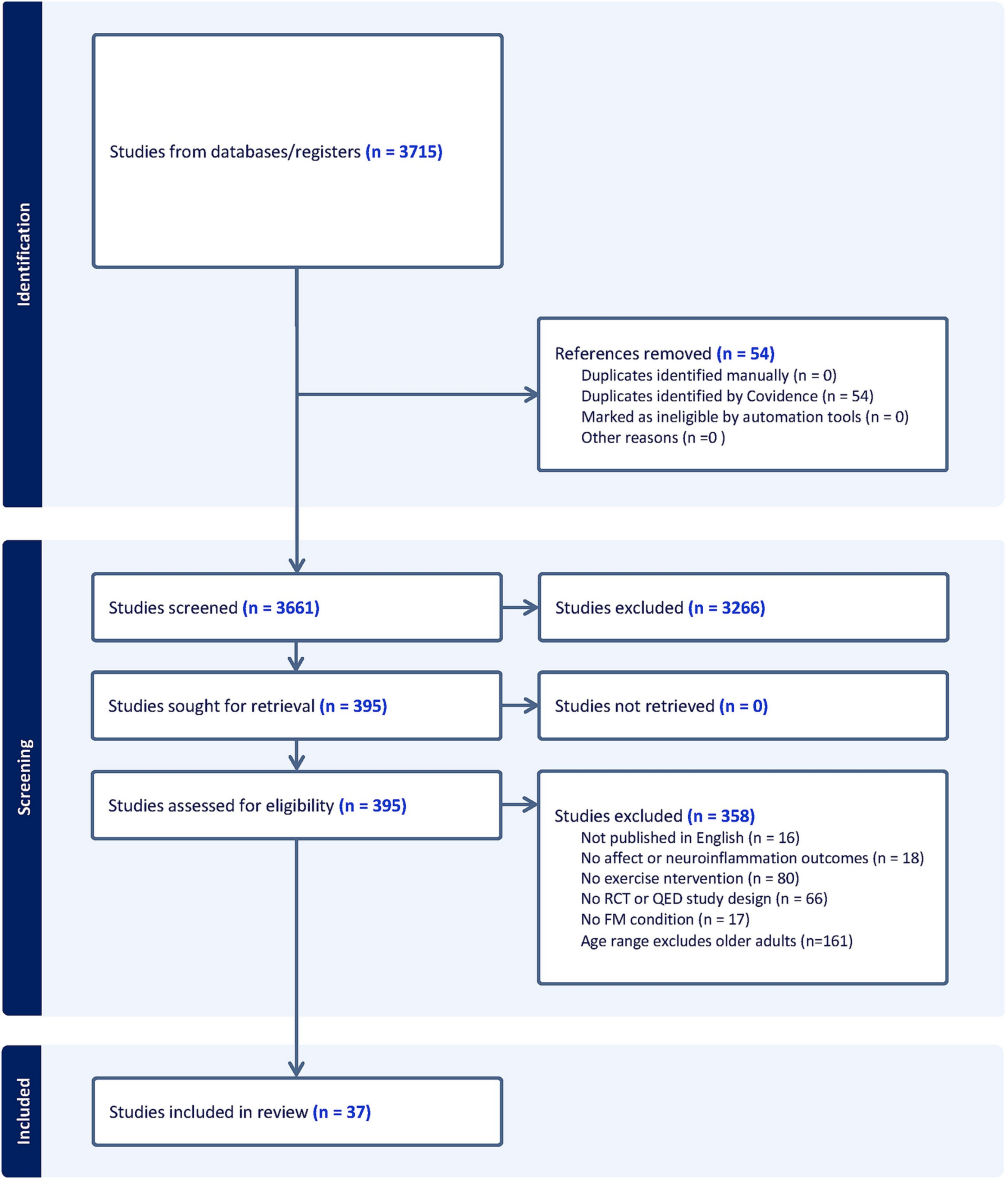
PRISMA flowchart.

Studies were identified examining the relationships among exercise, affect, neuroinflammation, and exercise adherence. Studies were screened based on PICOS (Population, Intervention, Comparator, Outcome, and Study Design) inclusion criteria that included: (1) sample includes older adults 65 years or older living with FM or a FM-related condition (i.e., Chronic Fatigue Syndrome); (2) an exercise intervention; (3) within or between group comparisons; (4) affect and/or neuroinflammation outcomes; and (5) a randomized controlled trial or quasi-experimental design. Exclusion criteria were: (1) no adults in the entire sample 65 years of age or older; (2) no FM or FM-related condition; (3) no exercise intervention; (4) no within or between comparisons; (5) no affect or neuroinflammation outcomes; and (6) observational, case-report, ongoing studies, books, or conference abstracts; and (7) full-text not available. See Figure 1 for articles excluded.

### 2.3 Search strategy and information sources

The University of Alabama at Birmingham Library conducted a comprehensive literature search in July 2023. Selected bibliographic databases included PubMed, CINAHL, Embase, CENTRAL, Academic Search Premier, SPORTDiscus, and PsychINFO. We searched for specific keywords and search terms included, but were not limited to, FM, affect, and neuroinflammation. See Supplementary material for more detailed search criteria conducted by the institution reference librarian. Abstracts were screened by five coauthors and studies that met eligibility were selected for full text review using Covidence review tool to manage search results.

### 2.4 Study records

Electronic database searches were imported to Covidence,^1^ where duplicates were automatically removed. Each abstract was screened by at least two reviewers with a total of six reviewers. Each article in full-text review was screened by at least two reviewers with a total of three reviewers. A standardized flow chart with PICOS criteria was used and any screening disagreements surrounding a specific study identified in Covidence were resolved through discussions about the relevance of the study’s inclusion criteria, study design, and/or outcome measures to our aims.

### 2.5 Data extraction

Data relating to review characteristics were extracted in Covidence and included publication type, study design, affect outcomes, neuroinflammation outcomes, exercise type, exercise adherence, and sample demographic information.

## 3 Results

### 3.1 Study characteristics

Studies spanned from 1994 to 2023 and the total number of FM participants undergoing exercise interventions across all studies was 2,466 (See Figure 1). All studies evaluated the use of exercise in FM samples. See Table 1 for extracted study characteristics of publication type, study design, affect outcomes, neuroinflammation outcomes, exercise type, exercise adherence and sample demographic information. Various exercise modalities were implemented. Fifteen studies implemented aerobic exercise, including stationary cycling, treadmill/outdoor walking and running, bodyweight-based exercises, aquatic exercises, and virtual reality workouts. Eleven studies utilized resistance exercise, 10 studies employed a combination of aerobic with resistance exercise, and five studies used stretching and flexibility exercises.

**Table 1:** Studies characteristics.

Author	Study design	Direction of effect of exercise on affect and neuroinflammation outcomes	Exercise type, mode, duration, frequency, and adherence	Demographic information
Arroyo-Fernández et al. (2022)	Randomized controlled trial	Depression ↓	Type: Aerobic Mode: N/A Duration: 1 h Frequency: 5 sessions Adherence: 97%	18–65 years (*n* = 120) FM 94% female; 6% male
Baptista et al. (2012)	Randomized controlled trial	Anxiety ↔ Depression ↔ Quality of life ↑	Type: Aerobic Mode: Belly dancing Duration: 1 h Frequency: 2 days a week for 16 weeks Adherence: 94%	18–65 years (*n* = 80) FM 100% female
Bodere et al. (2020)	Quasi-experimental design	Anxiety ↓ Depression ↓ Quality of life ↑	Type: Aerobic Mode: Biking, elliptical, and treadmill Duration: 45 min Frequency: 3 days a week Adherence: 65%	18–74 years (*n* = 138) FM 100% female
Calandre et al. (2009)	Randomized controlled trial	Anxiety ↔ Depression ↓ Quality of life ↑	Type: Stretching and flexibility Mode: Tai Chi Duration: 40 min Frequency: 3 days a week for 6 weeks Adherence: N/A	28–69 years (*n* = 81) FM 90% female; 10% male
Corrales et al. (2010)	Randomized controlled trial	Depression ↑ Quality of life ↑	Type: Aerobic Mode: Treadmill Duration: N/A Frequency: 2 days a week for 24 weeks Adherence: N/A	52–66 years (*n* = 42) FM 100% female
Ernberg et al. (2016)	Randomized controlled trial	IL-1Î^2^ ↔ TNF ↔ IL-6 ↔ IL-8 ↔	Type: Resistance Mode: N/A Duration: 60 min Frequency: 2 days a week for 15 weeks Adherence: N/A	48–66 years (*n* = 24) FM 100% female
Espí-López et al. (2016)	Randomized controlled trial	Depression ↓ Quality of life ↑	Type: Combined aerobic and resistance Mode: N/A Duration: 60 min Frequency: 2 days a week for 8 weeks Adherence: 74%	30–80 years (*n* = 35) FM 94% female; 6% male
Franco et al. (2023)	Randomized controlled trial	Quality of life ↑	Type: Combined aerobic and resistance Mode: Treadmill and bicycle Duration: 60 min Frequency: 2 days a week for 8 weeks Adherence: 90%	20–75 years (*n* = 97) FM 98% female; 2% male
Gandhi et al. (2002)	Quasi-experimental design	Quality of life ↑	Type: Combined aerobic and resistance Mode: N/A Duration: 1 h and 30 min Frequency: 2 days a week for 10 weeks Adherence: 69%	28–65 years (*n* = 32) FM 100% female
Glasgow et al. (2017)	Randomized controlled trial	Quality of life ↑	Type: Resistance Mode: Chest press, leg extension, leg curl, and seated row Duration: 30 min Frequency: 2 days a week for 8 weeks Adherence: 96%	19–65 years (*n* = 26) FM 100% female
Gusi et al. (2006)	Randomized controlled trial	Anxiety ↓ Depression ↓	Type: Aerobic Mode: Walking in a pool duration: 60 min Frequency: 3 days a week for 12 weeks Adherence: 97%	35–73 years (*n* = 34) FM 100% female
Haak and Scott (2008)	Randomized controlled trial	Anxiety ↓ Depression ↓ Quality of life ↑	Type: Stretching and flexibility Mode: Meditation Qi Gong Duration: N/A Frequency: 9 days over 7 weeks Adherence: 93%	27–73 years (*n* = 57) FM 100% female
Hernando-Garijo et al. (2021)	Randomized controlled trial	Anxiety ↓ Depression ↓ Quality of life ↑	Type: Aerobic Mode: Telerehabilitation Duration: 50 min Frequency: 2 days a week for 15 weeks Adherence: 83%	30–75 years (*n* = 34) FM 100% female
Izquierdo-Alventosa et al. (2021)	Randomized controlled trial	Quality of life ↑	Type: Combined aerobic and resistance Mode: Walking, upper and lower limb exercises Duration: 60 min Frequency: 2 days a week for 8 weeks Adherence: 100%	30–70 years (*n* = 49) FM 100% female
Jablochkova et al. (2019)	Randomized controlled trial	Anxiety ↔ Depression ↔ Quality of life ↔ NGF ↔ BDNF ↔	Type: Resistance Mode: Lower body strength training Duration: 50 min Frequency: 2 days a week for 15 weeks Adherence: N/A	20–65 years (*n* = 75) FM 100% female
Jones et al. (2008)	Randomized controlled trial	Anxiety ↓ Depression ↓ Quality of life ↔	Type: Combined aerobic and resistance Mode: Cardioaerobics, strength, flexibility, balancing elastic bands, and weights Duration: 60 min Frequency: 3 days a week for 6 months Adherence: 93.3%	18–65 years (*n* = 165) FM 97% female; 3% male
Kaleth et al. (2013)	Randomized controlled trial	Quality of life ↓	Type: Aerobic Mode: N/A Duration: 10–12 min increased to 28–30 min Frequency: 2–3 days a week increased to 3–4 days a week Adherence: 78.7%	18–65 years (*n* = 170) FM 95% female; 5% male
Kas et al. (2016)	Quasi-experimental design	Quality of life ↑	Type: Combined aerobic and resistance Mode: Upper and lower extremity exercises Duration: 60 min Frequency: 1–2 days a week for 10 weeks Adherence: N/A	18–65 years (*n* = 79) FM Females and males % not specified
Kibar et al. (2015)	Randomized controlled trial	Depression↓ Quality of life ↑	Type: Stretching and flexibility Mode: 2-Legged stand, semi-tandem stand, tandem stand, 1-legged stand, tandem walk, circle turns, heel or toe stands, standing with eyes closed Duration: 20 min Frequency: 5 days a week for 4 weeks Adherence: 84%	18–65 years (*n* = 57) FM 95% female; 5% male
López-Pousa et al. (2015)	Randomized controlled trial	Quality of life ↑	Type: Aerobic Mode: Walk Duration: N/A Frequency: 6 days Adherence: N/A	52–68 years (*n* = 30) FM 100% female
Maestre-Cascales et al. (2022)	Quasi-experimental design	Quality of life ↑	Type: Resistance Mode: Strength training Duration: 1 h Frequency: 2 days a week for 24 weeks Adherence: 100%	20–75 years (*n* = 41) FM 100% female
Matias et al. (2022)	Randomized controlled trial	Anxiety↔ Depression↔ Quality of life ↑	Type: Combined aerobic and resistance Mode: N/A Duration: 40 min Frequency: 3 days a week for 8 weeks Adherence: N/A	30–70 years (*n* = 31) FM 100% female
Nichols and Glenn (1994)	Quasi-experimental design	Quality of life ↑	Type: Aerobic Mode: Walking Duration: 20 min Frequency: 3 days per week for 8 weeks Adherence: N/A	30–69 years (*n* = 19) FM 89% female; 11% male
Park et al. (2021)	Randomized controlled trial	Quality of life ↑	Type: Resistance Mode: N/A Duration: 30 min Frequency: 2 days a week for 4 weeks Adherence: N/A	35–65 years (*n* = 40) FM 95% female; 5% male
Rodríguez-Mansilla et al. (2023)	Randomized controlled trial	Quality of life ↑	Type: Stretching and flexibility Mode: Qi Gong Duration: 45 min Frequency: 2 days a week for 4 weeks Adherence: N/A	30–65 years (*n* = 141) FM 100% female
Rooks et al. (2007)	Randomized controlled trial	Anxiety ↓ and ↔ Depression ↓ and ↔ Quality of life ↑ and ↔	Type: Aerobic or combined aerobic and resistance Mode: Treadmill walking or treadmill walking and strength training Duration: 60 min Frequency: 2 days a week for 16 weeks Adherence: 73%	18–75 years (*n* = 207) FM 100% female
Salm et al. (2019)	Randomized controlled trial	Anxiety ↔ Depression ↔ Quality of life ↔	Type: Aerobic Mode: Aquatic Duration: 18–50 min Frequency: 3 days a week for 6 weeks Adherence: 90%	30–69 years (*n* = 28) FM 100% female
Sañudo et al. (2011)	Randomized controlled trial	Depression ↔ Quality of life ↑	Type: Aerobic Mode: Walking and jogging Duration: 1 h Frequency: 2 days a week for 24 weeks Adherence: 85%	18–65 years (*n* = 42) FM 100% female
Sarmento et al. (2020)	Randomized controlled trial	Anxiety ↓ Depression ↓ Quality of life ↑	Type: Stretching and flexibility Mode: Qigong Duration: 25 min Frequency: 2 days a week for 10 weeks Adherence: N/A	18–70 years (*n* = 20) FM 100% female
Sauch Valmaña et al. (2020)	Randomized controlled trial	Quality of life ↔	Type: Combined aerobic and resistance Mode: N/A Duration: 1 h and 30 min Frequency: 2 days a week for 12 weeks Adherence: N/A	40–75 years (*n* = 24) FM 100% female
Sousa et al. (2023)	Randomized controlled trial	Quality of life ↑	Type: Aerobic Mode: Aquatic Duration: 45 min Frequency: 2 days a week for 12 weeks Adherence: N/A	18–70 years (*n* = 75) FM 100% female
Stensson et al. (2020)	Quasi-experimental design	Anxiety ↔ Depression ↓ Quality of life ↑	Type: Resistance Mode: Weights Duration: 60 min Frequency: 2 days a week for 15 weeks Adherence: N/A	20–65 years (*n* = 24) FM 100% female
Tomas-Carus et al. (2008)	Randomized controlled trial	Anxiety ↓ Depression ↓ Quality of life ↑	Type: Aerobic Mode: Pool Exercise Duration: 1 h Frequency: N/A Adherence: N/A	37–71 years (*n* = 30) FM 100% female
Toprak Celenay et al. (2017)	Randomized controlled trial	Anxiety ↓ Depression ↔ Quality of life ↑	Type: Combined aerobic and resistance Mode: Treadmill walking and strength exercises Duration: 1 h Frequency: 2 days a week for 6 weeks Adherence: N/A	18–65 years (*n* = 56) FM 100% female
Torres Vilarino et al. (2022)	Randomized controlled trial	Anger ↔ Depression ↔	Type: Resistance Mode: N/A Duration: 45 min to 1 h Frequency: 2 days a week for 8 weeks Adherence: N/A	18–70 years (*n* = 38) FM 100% female
Villafaina et al. (2019)	Randomized controlled trial	Quality of life ↑	Type: Aerobic Mode: Virtual exergame Duration: 1 h Frequency: 2 days a week for 24 weeks Adherence: N/A	30–75 years (*n* = 56) FM 100% female
Wåhlén et al. (2022)	Quasi-experimental design	Depression ↓ Quality of life ↔ P80723 ↔	Type: Resistance Mode: N/A Duration: 60 min Frequency: 2 days a week for 15 weeks Adherence: N/A	20–65 years (*n* = 40) FM 100% female

↓, significant decrease; ↑, significant increase; ↔, no change; N/A, not available; IL, Interleukin; TNF, Tumor necrosis factor; NGF, Nerve growth factor; BDNF, Brain derived neurotrophic factor; P, Protein.

### 3.2 Affect outcomes

All studies, except one, evaluated the influence of exercise on affect-related outcomes such as self-reported anxiety, depression, quality of life, and anger (Ernberg et al., 2016; Franco et al., 2023; Glasgow et al., 2017; Jablochkova et al., 2019; Maestre-Cascales et al., 2022; Park et al., 2021; Rooks et al., 2007; Stensson et al., 2020; Torres Vilarino et al., 2022; Wåhlén et al., 2022). Out of the 10 studies that included resistance exercise, mixed results were presented from four studies on depression, three studies on anxiety, and nine studies on quality of life with no studies revealing an influence of resistance exercise on anger. One of the four studies found a decrease in depression, while three found no change. Furthermore, two studies found no change on anxiety, and one found a decrease. For quality of life, four studies did not observe any changes with a decrease of the impact of FM.

Nine studies examined combined influence of resistance and aerobic exercise (Arroyo-Fernández et al., 2022; Espí-López et al., 2016; Gandhi et al., 2002; Izquierdo-Alventosa et al., 2021; Jones et al., 2008; Kas et al., 2016; Matias et al., 2022; Sauch Valmaña et al., 2020; Toprak Celenay et al., 2017). Six studies suggested decreased impact of FM on quality of life, two studies had no change. Depression decreased in three studies and did not change in two. Anxiety decreased in two studies and did not change in one.

Thirteen studies examined the influence of aerobic exercise only on affect (Baptista et al., 2012; Bodere et al., 2020; Corrales et al., 2010; Gusi et al., 2006; Hernando-Garijo et al., 2021; Kaleth et al., 2013; López-Pousa et al., 2015; Nichols and Glenn, 1994; Salm et al., 2019; Sanudo et al., 2011; Sousa et al., 2023; Tomas-Carus et al., 2008; Villafaina et al., 2019). Anxiety decreased in four studies, while no change in two. Depression decreased in four, increased in one, and did not change in three. Whereas impact on quality of life decreased in seven, increased in one, and did not change in one.

Subsequently, five studies assessed stretching and flexibility exercise influences on affect (Calandre et al., 2009; Haak and Scott, 2008; Kibar et al., 2015; Rodríguez-Mansilla et al., 2023; Sarmento et al., 2020). All improved measures of quality of life, four studies found decreases in depression, two studies found decreases in anxiety while one found no change.

### 3.3 Neuroinflammation outcomes

Three studies implementing resistance exercise effects on circulating neuroinflammation related markers found no changes to any of their outcomes including NGF, BDNF, P80723, L-1Î^2^, TNF, IL-6, and IL-8 (Ernberg et al., 2016; Jablochkova et al., 2019; Wåhlén et al., 2022).

### 3.4 Exercise adherence

Nineteen studies reported exercise adherence as a secondary outcome with adherence rates ranging from 65 to 100%. Study duration varied from one session to 24 weeks. Adherence was reported descriptively in two ways: as a percentage of sessions attended or as the proportion of participants who completed the full program.

## 4 Discussion

Our findings support the use of exercise as a tool to improve affect and affect-related outcomes, however, there is limited evidence for exercise effects on neuroinflammation or inflammation outcomes in older individuals living with FM. Additionally, we found that all types of exercise produced benefits across affect-related outcomes. However, preliminary evidence from our literature search suggests various types of exercise may differentially influence affect and neuroinflammation outcomes. Exercise-induced alterations of affect and neuroinflammation have implications for pain management in older adults living with FM and offer a cost-effective way to improve quality of life. It is noted that in many cases, the potential attribution of beneficial effects to affect and neuroinflammation remains speculative because of the lack of definitive data in this area. However, evidence regarding adherence during exercise is limited and heterogeneous within studies of people living with FM.

### 4.1 Affect benefits of exercise

Exercise is widely demonstrated to have a positive impact on mood and emotion, with numerous studies underscoring its benefits across various age groups excluding older adults. Regular physical activity significantly reduces symptoms of depression and anxiety, attributed to the release of endorphins and other neurotransmitters that enhance mood (Rebar et al., 2015; Schuch et al., 2016). Furthermore, exercise improves cognitive function and emotional regulation by increasing hippocampal volume and promoting neurogenesis (Deodato et al., 2024; Erickson et al., 2011; Mellow et al., 2020; Morris et al., 2020; Voss et al., 2011). Beyond its neurobiological effects, exercise also facilitates social interaction and self-efficacy, crucial for emotional wellbeing. Participating in group activities can reduce feelings of loneliness and social anxiety (Dunn et al., 2005). Achieving fitness goals enhances self-esteem, further boosting emotional health (Biddle and Asare, 2011). The psychological benefits of exercise are well-documented across various forms, highlighting its versatility as a non-pharmacological intervention for mood enhancement (Meyer et al., 2016; Wegner et al., 2014).

Our literature review revealed a large research gap regarding the efficacy of exercise at improving affect-related outcomes specifically among older adults with chronic pain conditions. As older adults comprise the majority of chronic pain populations, future research is warranted to broaden the existing knowledge on the association between exercise habits and affect. We focused on investigating exercise-induced changes in affect-related outcomes among older adults living with FM and FM-related conditions, rather than relying solely on cross-sectional comparisons between active and sedentary groups. Within this scoping review we primarily examined the impact on depression, anxiety, and quality of life. We found that all types of exercise including aerobic and resistance exercise, combined exercise, Qi Gong, walking, and daily leisure exercise produced significant affective benefits for older adults. However, it is important to note that depression, anxiety, and quality of life measures were the only outcomes consistently reported across the studies analyzed. Whether habitual exercise is capable of attenuating chronic pain via modifications in affect balance style remains unknown. While exercise alters state and trait affect, the magnitude of change in affect balance style clusters is yet to be determined. Addressing this gap in knowledge is crucial for advancing our understanding of the interplay between exercise, affect, and chronic pain in older adults.

### 4.2 Neuroinflammation benefits of exercise

Neuroinflammation is associated with both chronic pain and depression, as demonstrated in imaging studies observing central nervous system glial activation in patients with either of these conditions (Albrecht et al., 2021; Holmes et al., 2018; Loggia et al., 2015). FM is characterized by both neuroinflammation and systemic inflammation, or stress-related chronic inflammation of the entire body (Backryd et al., 2017). For instance, a study assessing a single cytokine in cerebrospinal fluid found evidence of neuroinflammation in FM (Kadetoff et al., 2012). Further, a follow-up study assessing a large array of inflammatory markers in cerebrospinal fluid demonstrated that neuroinflammation is upregulated simultaneously in individuals living with FM compared to healthy controls (Backryd et al., 2017). Additionally, systemic inflammation is associated with clinical pain symptoms in other chronic pain conditions (Merlin et al., 2017). It is hypothesized that these markers yield a pain-processing imbalance, which places individuals on a spectrum between anti-nociception, the body’s response to harm, injury, and extreme temperature and pro-nociception, the body’s facilitated pain processing and/or reduced pain-modulatory capabilities (Cheng et al., 2015; Qadir et al., 2020; Yarnitsky et al., 2014).

While neuroinflammation has emerged as a promising target for interventions in individuals living with FM, studies exploring the relationship between negative affect, pain, and neuroinflammation are limited. Additionally, imaging studies exploring neuroinflammation as a modifiable target in the management of FM pain are also limited. Subsequently, existing interactions are present between central nervous system mediators and peripheral inflammatory markers (Kadetoff et al., 2012). Resistance exercise offers potential in managing inflammatory markers indicative of neuroinflammation, but more studies are recommended to examine how different modes of exercise, volume, and duration may affect neuroinflammation and circulating inflammatory markers. Of importance, aerobic and resistance exercise alleviates these markers in other neuroinflammation-related diseases, such as Alzheimer’s disease, Parkinson’s disease, amyotrophic lateral sclerosis, and multiple sclerosis (Seo et al., 2019). To elucidate the cellular and/or molecular mechanisms that underlie the role of exercise in attenuating the activation of microglia and pro-inflammatory cytokines in the brain, further clinical and preclinical studies are suggested for accurate testing. Recent work reporting reductions in inflammatory markers following aerobic and resistance exercise in healthy middle age and older adults (Zheng et al., 2019). Therefore, findings from this review are inconclusive and interpreted with caution.

### 4.3 Exercise adherence considerations for older adults living with FM

Previous research has established that regular, long-term exercise programs can be beneficial to the wellbeing and functionality of individuals living with FM. Low exercise compliance and high dropout rates in the short or long term is well-documented in individuals living with FM (Sarmento et al., 2023). Exercise can result in an immediate worsening of pain and fatigue in individuals living with FM, challenging their exercise program tolerance. In a narrative literature review exploring adherence to moderate-to-high-intensity exercises in patients living with FM, it was reported that exercise programs worsened pain and fatigue symptoms, which led to high dropout rates and low exercise adherence rates (Sarmento et al., 2023). High dropout rates were reported in home-based or self-paced exercise programs, and patients prefer supervised exercise, which, if implemented, could increase adherence. Identifying and overcoming barriers associated with the initiation and maintenance of a supervised center-based exercise program is critical to the successful management of pain symptoms. More studies are needed to further investigate the connection between individual patient characteristics and dropout from an exercise program.

Exercise is commonly recommended to manage pain symptoms, but exercise engagement and adherence are particularly low among populations with chronic pain. Along with pain intensity, evidence suggests older adults perceive stress or depression as major barriers to engagement in regular exercise (Busch et al., 2008). Co-morbid depression and stress are often cited in research as barriers to exercise programs and are associated with exercise avoidance (Collado-Mateo et al., 2021; Lazaridou et al., 2020). Recent work has applied the NIA Health Disparities Framework to identify priority areas of need in pain research; specifically, highlighting geographic and political factors, cultural factors, coping factors, and physiological indicators as priority focus areas (Patel et al., 2022). Therefore, designing patient-informed exercise programs as a pain management tool among older adults living with FM could potentially increase exercise adherence. Ensuring that future programs designed to target exercise barriers are feasible, accessible, and effective is vital to enhance the therapeutic benefits of exercise for older adults living with FM.

### 4.4 Clinical and research implications

**Figure 2:**
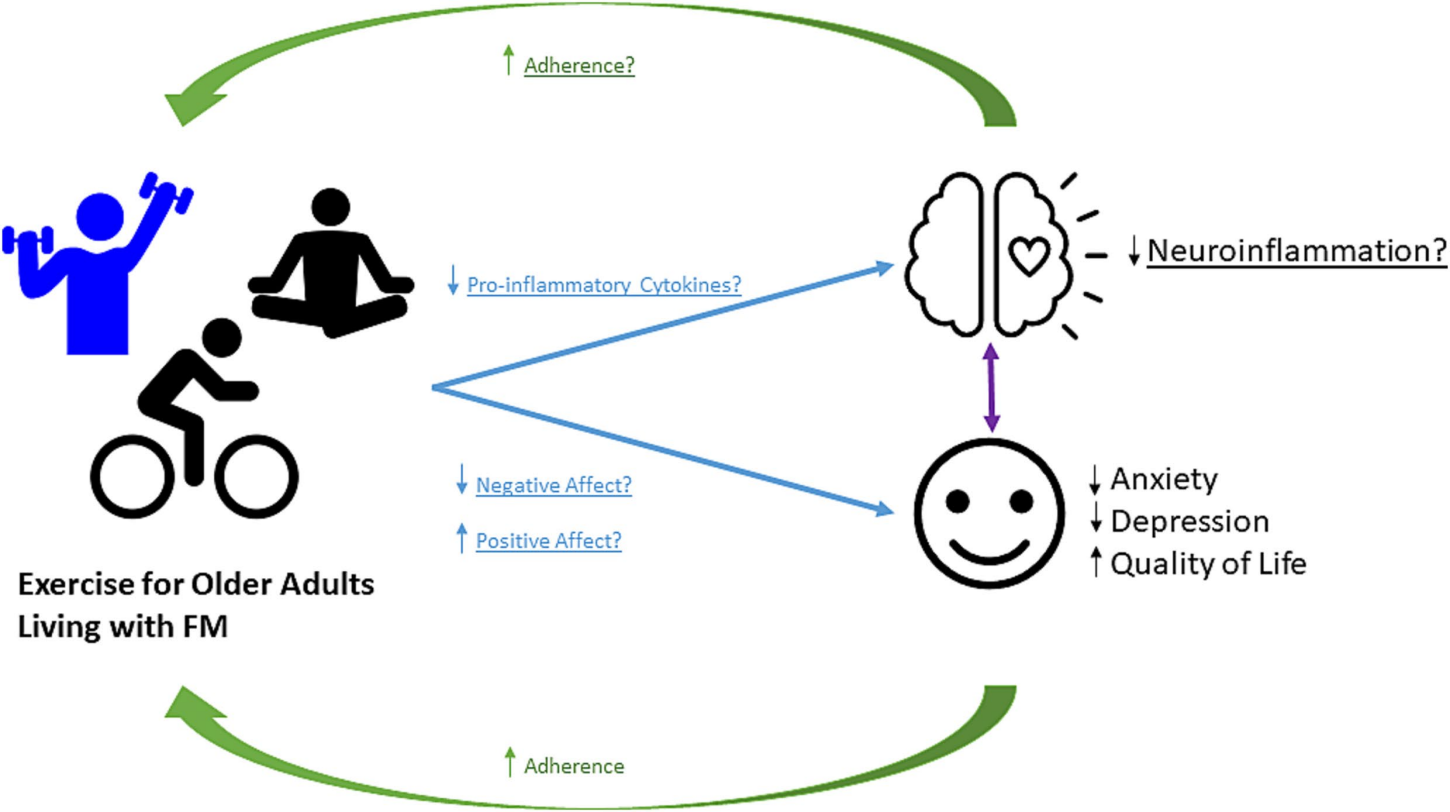
Conceptual Framework. Proposed framework for how exercise alters the affect and neuroinflammation in older adults living with FM and with potential implications for exercise adherence. Exercise increases positive affect, and decreases negative affect and pro-inflammatory cytokines. Exercise provides benefits to pain outcomes in individuals living with FM. Whether this disease protection is mediated by exercise-induced changes in affect and neuroinflammation remains to be determined.

Clinicians and researchers can benefit greatly from considering the emotional and physical benefits of prescribing exercise, while also recognizing the barriers to adherence for older adults living with FM. Though under-recognized to date, many of the benefits are apparent via interactions with affect and neuroinflammation. Here, we discuss example conditions for which the affect and neuroinflammation may play a pivotal role, though they almost certainly do not represent the full spectrum of potential benefits. Based on our findings and gaps that remain, we propose a conceptual framework that illustrates the benefits of exercise, proposed mechanisms, and implications for adherence (See Figure 2). We have summarized evidence of the beneficial effects of exercise on anxiety (Bodere et al., 2020; Gusi et al., 2006; Haak and Scott, 2008; Hernando-Garijo et al., 2021; Jones et al., 2008; Rooks et al., 2007; Sarmento et al., 2020; Tomas-Carus et al., 2008; Toprak Celenay et al., 2017), depression (Arroyo-Fernández et al., 2022; Bodere et al., 2020; Calandre et al., 2009; Espí-López et al., 2016; Gusi et al., 2006; Haak and Scott, 2008; Hernando-Garijo et al., 2021; Jones et al., 2008; Kibar et al., 2015; Rooks et al., 2007; Sarmento et al., 2020; Stensson et al., 2020; Tomas-Carus et al., 2008; Wåhlén et al., 2022), and quality of life (Baptista et al., 2012; Bodere et al., 2020; Calandre et al., 2009; Corrales et al., 2010; Espí-López et al., 2016; Franco et al., 2023; Gandhi et al., 2002; Glasgow et al., 2017; Haak and Scott, 2008; Hernando-Garijo et al., 2021; Izquierdo-Alventosa et al., 2021; Kaleth et al., 2013; Kas et al., 2016; Kibar et al., 2015; López-Pousa et al., 2015; Maestre-Cascales et al., 2022; Matias et al., 2022; Nichols and Glenn, 1994; Park et al., 2021; Rodríguez-Mansilla et al., 2023; Rooks et al., 2007; Sanudo et al., 2011; Sarmento et al., 2020; Sousa et al., 2023; Stensson et al., 2020; Tomas-Carus et al., 2008; Toprak Celenay et al., 2017; Villafaina et al., 2019). While we did not see changes in inflammatory and neuroinflammatory markers, the number of studies was very limited. Therefore, in Figure 2, based on impact of exercise on these factors in other pain and aging populations, we propose that it is possible that changes in pro-inflammatory cytokines, positive affect, and negative affect may contribute to benefits in affect-related and neuroinflammation-related outcomes (Hassett et al., 2008; Seo et al., 2019; Zheng et al., 2019). These beneficial effects are also related to the management of pain and identify barriers to exercise adherence. However, as we have found in this review there are still gaps remaining that are critical to address moving forward. By addressing these areas, future work can improve outcomes related to FM-pain, and further address considerations for older adults living with FM to increase exercises adherence and pain management, and emotional and physical quality of life in these individuals (Mueller et al., 2020; Troubat et al., 2021).

### 4.5 Strengths and limitations

Several strengths and limitations exist within this scoping review. First, we used a comprehensive search strategy to identify studies reporting the most investigated exercise interventions for FM and their major outcomes. However, observational study designs and secondary analyses of the data were not included in our search since we were focused on effects of exercise interventions. While we attempted a comprehensive scoping review via including at least two reviewers per article, searching multiple databases, and consulting with institutional library services on search strategy. Secondly, we discovered most of the participants in the included articles comprised of young and middle-aged adults, in addition to older adults. While recent literature highlights prevalence and significance of FM in older adults, the literature is lacking in exercise interventions specifically recruiting older adults (Benlidayi, 2023). Additionally, our review provided an initial comprehensive summary of the hypothesized mechanisms for affect and neuroinflammatory effects of exercise in older adults living with FM. Therefore, more studies are needed to examine how exercise works as a mechanism to improve neuroinflammation specifically for older adults living with FM only. Due to the current study being a scoping review, all available literature was included regardless of quality assessments and therefore study conclusions were not weighted but allowed us to provide a broader overview of this area. Quality assessments were conducted in Covidence. Nine studies had high risk of bias in sequence generation, 11 studies had high risk in allocation concealment, 18 had high risk in blinding participants and personnel, 15 had high risk in blinding of outcome, 18 had high risk in missing data. All studies had low risk of bias in selective reporting and other biases.

### 4.6 Conclusion and future directions

In conclusion, this scoping review underscores the potential of exercise to impact affective states and neuroinflammatory markers in older adults coping with FM, potentially leading to enhanced exercise adherence. Despite these promising indications, the available evidence remains limited and current evidence on the topic remains debated. Consequently, there is a critical need for further research aimed at standardizing exercise protocols while considering both emotional and physical factors specific to older adults. Further, exploring the implications of exercise-induced changes in affect and neuroinflammation for pain management in FM patients will deepen our understanding of effective pain management strategies. Studying the relationship between exercise, affect, and neuroinflammation may provide valuable insights on how to optimally engage older adults living with FM and improve quality of life. Expanding our knowledge of the role of exercise in modulating these mechanisms is essential not only for FM patients but also for individuals managing chronic inflammatory and musculoskeletal conditions. By gaining a deeper understanding of these interactions, we can empower individuals to manage their chronic pain and improve their overall wellbeing. Thus, continued research in this area is vital for advancing both clinical practice and patient outcomes.

Our review provides several potential directions for future research on exercise and FM. Overall findings from the current review highlight the need for future work that will assess the contribution of affect and neuroinflammation to exercise adherence for individuals living with FM. The identification of exercise responses based off psychophysiological mechanisms will provide a basis for future work validating these as possible biomarkers of FM. To this end, future studies should also explore the relationship between exercise responses with affect and neuroinflammation in other FM-related conditions. Other work includes clarifying short-term and long-term exercise related changes in affect and neuroinflammation. Lastly, a need exists to also promote exercise adherence through qualitative approaches. Thus, a future fully powered trial could look at various exercise types and collect feedback on the exercise interventions from participants, to address FM-related barriers to exercise engagement and further focus on age-related declines in quality of life that can be further exacerbated by FM pain and the biopsychosocial factors contributing to their pain (e.g., affect and neuroinflammation).

## Data Availability

The raw data supporting the conclusions of this article will be made available by the authors, without undue reservation.

## Footnotes

^1^ https://www.covidence.org
