# Supplementary material for: The potential impact of exercise on affect and neuroinflammation in older adults living with fibromyalgia: a scoping review
Source: Front Hum Neurosci. 2025 Jan 6;18:1463935. doi: 10.3389/fnhum.2024.1463935 (PMC11743465; doi:10.3389/fnhum.2024.1463935)
Supplement: Supplementary file 1 [file Table_1.DOCX]

**Supplement 1:** Example of the search strategies for all databases

*PubMed*

("Fibromyalgia"[Mesh] OR "Fatigue Syndrome, Chronic"[Mesh] OR Fibromyalgia*[Title/Abstract] OR Muscular-Rheumatism*[Title/Abstract] OR Fibrositis[Title/Abstract] OR Fibrositides[Title/Abstract] OR Diffuse-Myofascial-Pain-Syndrome*[Title/Abstract] OR fibro[Title/Abstract] OR Chronic-Fatigue[Title/Abstract] OR Systemic-Exertion-Intolerance-Disease*[Title/Abstract] OR Myalgic-Encephalomyelitis[Title/Abstract] OR Postviral-Fatigue[Title/Abstract] OR Chronic-Infectious-Mononucleosis-Like[Title/Abstract] OR Royal-Free[Title/Abstract] OR Musculoskeletal-Pain-Syndrome[Title/Abstract] OR Myofibrositis[Title/Abstract] OR Fibromyositis[Title/Abstract])

AND

("Adult"[Mesh] OR adult*[Title/Abstract] OR elder*[Title/Abstract] OR aged [Title/Abstract] OR geriatric*[Title/Abstract] OR gerontol* [Title/Abstract] OR College-student* [Title/Abstract] OR university-student* [Title/Abstract] OR men [Title/Abstract] OR women [Title/Abstract] OR man [Title/Abstract] OR woman [Title/Abstract])

AND

("Inflammation"[Mesh] OR "Microglia"[Mesh] OR "Cytokines"[Mesh] OR "Transforming Growth Factors"[Mesh] OR "Astrocytes"[Mesh] OR "HLA Antigens"[Mesh] OR "Histocompatibility Antigens Class II"[Mesh] OR "Histocompatibility Antigens Class I"[Mesh] OR "Cerebrospinal Fluid"[Mesh] OR "C-Reactive Protein"[Mesh] OR "Brain-Derived Neurotrophic Factor"[Mesh] OR "Glutamate Plasma Membrane Transport Proteins"[Mesh] OR "N-acetylaspartate" [Supplementary Concept] OR "myo-inositol 1,3,5-orthobutyrate" [Supplementary Concept] OR "Emotions"[Mesh] OR "Stress, Psychological"[Mesh] OR "Anxiety"[Mesh] OR "Quality of Life"[Mesh] OR neuroinflammat* [title/abstract] OR inflammat* [title/abstract] OR Microglia*[Title/Abstract] OR proinflammatory[Title/Abstract] OR pro-inflammatory[Title/Abstract] OR cytokine*[Title/Abstract] OR Intercrine* [tiab] OR chemokine*[Title/Abstract] OR interleukin*[Title/Abstract] OR IL-6[Title/Abstract] OR IL6[Title/Abstract] OR B-Cell Stimulatory-Factor-2[Title/Abstract] OR B-Cell-Differentiation-Factor-2[Title/Abstract] OR BSF-2[Title/Abstract] OR Hybridoma-Growth-Factor*[Title/Abstract] OR Plasmacytoma-Growth-Factor*[Title/Abstract] OR Hepatocyte-Stimulating-Factor*[Title/Abstract] OR MGI-2[Title/Abstract] OR Myeloid-Differentiation-Inducing-Protein*[Title/Abstract] OR IL-8[Title/Abstract] OR IL8[Title/Abstract] OR Monocyte-Derived-Neutrophil-Chemotactic-Factor*[Title/Abstract] OR Neutrophil-Activation-Factor*[Title/Abstract] OR Lymphocyte-Derived-Neutrophil-Activating-Peptide*[Title/Abstract] OR Monocyte-Derived-Neutrophil-Activating-Peptide*[Title/Abstract] OR Alveolar-Macrophage-Chemotactic-Factor-I[Title/Abstract] OR AMCF-I[Title/Abstract] OR Anionic-Neutrophil-Activating-Peptide*[Title/Abstract] OR Macrophage-Derived-Chemotactic-Factor*[Title/Abstract] OR Neutrophil-Chemotactic-Factor*[Title/Abstract] OR Granulocyte-Chemotactic-Peptide-Interleukin-8[Title/Abstract] OR IL-1[Title/Abstract] OR T-Helper-Factor*[Title/Abstract] OR Lymphocyte-Activating-Factor*[Title/Abstract] OR Macrophage-Cell-Factor*[Title/Abstract] OR Epidermal-Cell-Derived-Thymocyte-Activating-Factor*[Title/Abstract] OR TNF[Title/Abstract] OR Tumor-necrosis-factor*[Title/Abstract] OR IFN[Title/Abstract] OR Interferon*[Title/Abstract] OR transforming-growth-factor*[Title/Abstract] OR TGF[Title/Abstract] OR microgliosis[Title/Abstract] OR astrocyt*[Title/Abstract] OR astrogli*[Title/Abstract] OR HLA[Title/Abstract] OR Human-leukocyte-antigen*[Title/Abstract] OR Leukocyte-antigen*[Title/Abstract] OR HL-A[Title/Abstract] OR Major-histocompatabilit*[Title/Abstract] OR MHC[Title/Abstract] OR MHC-I [title/abstract] OR Immune-response-antigen*[Title/Abstract] OR Immune-response-associated-antigen*[Title/Abstract] OR I-A-Antigen*[Title/Abstract] OR Ia-Like-Antigen*[Title/Abstract] OR Class-II-MHC-Protein*[Title/Abstract] OR Class-II-Antigen*[Title/Abstract] OR Class-II-Histocompatibility-Antigen*[Title/Abstract] OR IA-Antigen*[Title/Abstract] OR Immune-Associated-Antigen*[Title/Abstract] OR MHC-Class-I[Title/Abstract] OR Class-I-Antigen*[Title/Abstract] OR Class-I-Histocompatibility-Antigen*[Title/Abstract] OR Class-I-MHC[Title/Abstract] OR Class-I-Antigen*[Title/Abstract] OR MHC-class-II[Title/Abstract] OR IL-4[Title/Abstract] OR B-Cell-Growth-Factor-1[Title/Abstract] OR B-Cell-Growth Factor-I[Title/Abstract] OR B-Cell-Proliferating-Factor*[Title/Abstract] OR B-Cell-Stimulating-Factor-1[Title/Abstract] OR B-Cell-Stimulatory-Factor-1[Title/Abstract] OR BCGF-1[Title/Abstract] OR Binetrakin*[Title/Abstract] OR BSF-1[Title/Abstract] OR IL4[Title/Abstract] OR Mast-Cell-Growth-Factor-2[Title/Abstract] OR MCGF-2[Title/Abstract] OR B-Cell-Stimulatory-Factor-1[Title/Abstract] OR CXC[Title/Abstract] OR C-X-C[Title/Abstract] OR alpha-Chemokine*[Title/Abstract] OR IL-10[Title/Abstract] OR IL10[Title/Abstract] OR CSIF-10[Title/Abstract] OR Cytokine-Synthesis-Inhibitory-Factor*[Title/Abstract] OR IL-12[Title/Abstract] OR IL12[Title/Abstract] OR Natural-Killer-Cell-Stimulatory-Factor*[Title/Abstract] OR Cytotoxic-Lymphocyte-Maturation-Factor*[Title/Abstract] OR Edodekin-Alfa[Title/Abstract] OR IL-11[Title/Abstract] OR IL11[Title/Abstract] OR Adipogenesis-Inhibitory-Factor*[Title/Abstract] OR Cachectin*[Title/Abstract] OR gamma-Interferon*[Title/Abstract] OR GM-CSFIL-4[Title/Abstract] OR Cerebrospinal-fluid*[Title/Abstract] OR Cerebro-Spinal-Fluid*[Title/Abstract] OR CX3CL1[Title/Abstract] OR Neurotactin*[Title/Abstract] OR Fractalkine*[Title/Abstract] OR CRP[Title/Abstract] OR c-reactive-protein*[Title/Abstract] OR hsCRP[Title/Abstract] OR BDNF[Title/Abstract] OR Brain-derived-neurotrophic-factor*[Title/Abstract] OR Glutamate-Specific-Neurotransmitter-Transporter*[Title/Abstract] OR Glutamate-Plasma-Membrane-Transporter-Protein*[Title/Abstract] OR Sodium-Glutamate-Cotransporter*[Title/Abstract] OR Sodium-Glutamate-Transporter*[Title/Abstract] OR Excitatory-Amino-Acid-Transporter-Protein*[Title/Abstract] OR Excitatory-Amino-Acid-Transport-Protein*[Title/Abstract] OR EAAT-Protein*[Title/Abstract] OR myo-inositol*[Title/Abstract] OR N-acetylaspartate[Title/Abstract] OR N-acetyl-L-aspartate[Title/Abstract] OR N-acetyl-aspartate[Title/Abstract] OR N-acetylaspartic-acid[Title/Abstract] OR Acetyl-aspartic-acid[Title/Abstract] OR tNAA[Title/Abstract] OR Emotion*[Title/Abstract] OR Valence[Title/Abstract] OR Arousal[Title/Abstract] OR Anxiet*[Title/Abstract] OR Stress*[Title/Abstract] OR Affect[Title/Abstract] OR Feel*[Title/Abstract] OR Mood*[Title/Abstract] OR Angst[Title/Abstract] OR Nervous*[Title/Abstract] OR Anxious*[Title/Abstract] OR Quality-of-life[Title/Abstract] OR Life-quality[Title/Abstract] OR Well-being[Title/Abstract] OR Wellness[Title/Abstract] OR Wellbeing[Title/Abstract] OR State[Title/Abstract] OR trait[Title/Abstract])

AND

("Sports"[Mesh] OR "Recreation"[Mesh] OR "Exercise"[Mesh] OR "Exercise Therapy"[Mesh] OR "Physical Exertion"[Mesh] OR "Exercise Movement Techniques"[Mesh] OR Exercise* [title/abstract] OR Plyometric* [title/abstract] OR Physical-Exertion* [title/abstract] OR physical-effort* [title/abstract] OR physical-activit*[title/abstract] OR Gymnastic*[title/abstract] OR Calisthenic*[title/abstract] OR stretch*[title/abstract] OR train*[title/abstract] OR run [title/abstract] OR runs [tiab] OR running [tiab] OR jog [title/abstract] OR jogs [tiab] OR jogging [tiab] OR swim*[title/abstract] OR walk*[title/abstract] OR climb*[title/abstract] OR weight-lift*[title/abstract] OR Ambulat*[title/abstract] OR Fitness[title/abstract] OR pilate*[title/abstract] OR qigong[title/abstract] OR qi-gong[title/abstract] OR ch’i-kung[title/abstract] OR danc*[title/abstract] OR tai-ji[title/abstract] OR tai-chi[title/abstract] OR tai-ji-quan[title/abstract] OR taiji[title/abstract] OR taijiquan[title/abstract] OR t’ai-chi[title/abstract] OR tai-chi-chuan[title/abstract] OR yoga[title/abstract] OR Sport*[title/abstract] OR athletic*[title/abstract] OR baseball*[title/abstract] OR softball*[title/abstract] OR basketball*[title/abstract] OR netball*[title/abstract] OR bicycling[title/abstract] OR cycling[title/abstract] OR boxing*[title/abstract] OR cricket[title/abstract] OR football*[title/abstract] OR rugb*[title/abstract] OR golf*[title/abstract] OR hockey*[title/abstract] OR wrestl*[title/abstract] OR martial-art*[title/abstract] OR hap-di-do[title/abstract] OR judo[title/abstract] OR karate[title/abstract] OR jujitsu[title/abstract] OR tae-kwon-do[title/abstract] OR aikido[title/abstract] OR wushu[title/abstract] OR kung-fu[title/abstract] OR gong-fu[title/abstract] OR gongfu[title/abstract] OR mountaineer*[title/abstract] OR tennis[title/abstract] OR Racquetball[title/abstract] OR Racketball[title/abstract] OR Racket-Ball[title/abstract] OR Badminton[title/abstract] OR Lacrosse[title/abstract] OR skating*[title/abstract] OR skateboard*[title/abstract] OR snowmobiling[title/abstract] OR sledding[title/abstract] OR skiing[title/abstract] OR snowboard*[title/abstract] OR soccer*[title/abstract] OR volleyball*[title/abstract] OR surfing[title/abstract] OR rowing[title/abstract] OR polo[title/abstract] OR kayaking[title/abstract] OR canoeing[title/abstract] OR boating[title/abstract] OR surfboarding[title/abstract] OR recreation*[title/abstract] OR garden*[title/abstract] OR strength* [title/abstract] OR resistance* [title/abstract])

*CINAHL*

( MH "Sports+" OR MH "Physical Activity" OR MH "Exercise+" OR MH "Recreation+" OR MH "Therapeutic Exercise+" OR MH "Exertion+" OR Exercise* OR Plyometric* OR Physical-Exertion* OR physical-effort* OR physical-activit* OR Gymnastic* OR Calisthenic* OR stretch* OR train* OR run OR runs OR running OR jog OR jogs OR jogging OR swim* OR walk* OR climb* OR weight-lift* OR Ambulat* OR Fitness OR pilate* OR qigong OR qi-gong OR ch’i-kung OR danc* OR tai-ji OR tai-chi OR tai-ji-quan OR taiji OR taijiquan OR t’ai-chi OR tai-chi-chuan OR yoga OR Sport* OR athletic* OR baseball* OR softball* OR basketball* OR netball* OR bicycling OR cycling OR boxing* OR cricket OR football* OR rugb* OR golf* OR hockey* OR wrestl* OR martial-art* OR hap-di-do OR judo OR karate OR jujitsu OR tae-kwon-do OR aikido OR wushu OR kung-fu OR gong-fu OR gongfu OR mountaineer* OR tennis OR Racquetball OR Racketball OR Racket-Ball OR Badminton OR Lacrosse OR skating* OR skateboard* OR snowmobiling OR sledding OR skiing OR snowboard* OR soccer* OR volleyball* OR surfing OR rowing OR polo OR kayaking OR canoeing OR boating OR surfboarding OR recreation* OR garden* OR strength* OR resistance* ) AND ( MH "Inflammation+" OR MH "Emotions+" OR MH "Glial Cells" OR MH "Cytokines+" OR MH "Histocompatibility Antigens+" OR MH "Cerebrospinal Fluid" OR MH "C-Reactive Protein" OR MH "Brain-Derived Neurotrophic Factor" OR MH "Stress, Psychological+" OR MH "Anxiety+" OR MH "Quality of Life+" OR MH "Psychological Well-Being" OR neuroinflammat* OR inflammat* OR Microglia* OR proinflammatory OR pro-inflammatory OR cytokine* OR Intercrine* OR chemokine* OR interleukin* OR IL-6 OR IL6 OR B-Cell Stimulatory-Factor-2 OR B-Cell-Differentiation-Factor-2 OR BSF-2 OR Hybridoma-Growth-Factor* OR Plasmacytoma-Growth-Factor* OR Hepatocyte-Stimulating-Factor* OR MGI-2 OR Myeloid-Differentiation-Inducing-Protein* OR IL-8 OR IL8 OR Monocyte-Derived-Neutrophil-Chemotactic-Factor* OR Neutrophil-Activation-Factor* OR Lymphocyte-Derived-Neutrophil-Activating-Peptide* OR Monocyte-Derived-Neutrophil-Activating-Peptide* OR Alveolar-Macrophage-Chemotactic-Factor-I OR AMCF-I OR Anionic-Neutrophil-Activating-Peptide* OR Macrophage-Derived-Chemotactic-Factor* OR Neutrophil-Chemotactic-Factor* OR Granulocyte-Chemotactic-Peptide-Interleukin-8 OR IL-1 OR T-Helper-Factor* OR Lymphocyte-Activating-Factor* OR Macrophage-Cell-Factor* OR Epidermal-Cell-Derived-Thymocyte-Activating-Factor* OR TNF OR Tumor-necrosis-factor* OR IFN OR Interferon* OR transforming-growth-factor* OR TGF OR microgliosis OR astrocyt* OR astrogli* OR HLA OR Human-leukocyte-antigen* OR Leukocyte-antigen* OR HL-A OR Major-histocompatabilit* OR MHC OR MHC-I OR Immune-response-antigen* OR Immune-response-associated-antigen* OR I-A-Antigen* OR Ia-Like-Antigen* OR Class-II-MHC-Protein* OR Class-II-Antigen* OR Class-II-Histocompatibility-Antigen* OR IA-Antigen* OR Immune-Associated-Antigen* OR MHC-Class-I OR Class-I-Antigen* OR Class-I-Histocompatibility-Antigen* OR Class-I-MHC OR Class-I-Antigen* OR MHC-class-II OR IL-4 OR B-Cell-Growth-Factor-1 OR B-Cell-Growth Factor-I OR B-Cell-Proliferating-Factor* OR B-Cell-Stimulating-Factor-1 OR B-Cell-Stimulatory-Factor-1 OR BCGF-1 OR Binetrakin* OR BSF-1 OR IL4 OR Mast-Cell-Growth-Factor-2 OR MCGF-2 OR B-Cell-Stimulatory-Factor-1 OR CXC OR C-X-C OR alpha-Chemokine* OR IL-10 OR IL10 OR CSIF-10 OR Cytokine-Synthesis-Inhibitory-Factor* OR IL-12 OR IL12 OR Natural-Killer-Cell-Stimulatory-Factor* OR Cytotoxic-Lymphocyte-Maturation-Factor* OR Edodekin-Alfa OR IL-11 OR IL11 OR Adipogenesis-Inhibitory-Factor* OR Cachectin* OR gamma-Interferon* OR GM-CSFIL-4 OR Cerebrospinal-fluid* OR Cerebro-Spinal-Fluid* OR CX3CL1 OR Neurotactin* OR Fractalkine* OR CRP OR c-reactive-protein* OR hsCRP OR BDNF OR Brain-derived-neurotrophic-factor* OR Glutamate-Specific-Neurotransmitter-Transporter* OR Glutamate-Plasma-Membrane-Transporter-Protein* OR Sodium-Glutamate-Cotransporter* OR Sodium-Glutamate-Transporter* OR Excitatory-Amino-Acid-Transporter-Protein* OR Excitatory-Amino-Acid-Transport-Protein* OR EAAT-Protein* OR myo-inositol* OR N-acetylaspartate OR N-acetyl-L-aspartate OR N-acetyl-aspartate OR N-acetylaspartic-acid OR Acetyl-aspartic-acid OR tNAA OR Emotion* OR Valence OR Arousal OR Anxiet* OR Stress* OR Affect OR Feel* OR Mood* OR Angst OR Nervous* OR Anxious* OR Quality-of-life OR Life-quality OR Well-being OR Wellness OR Wellbeing OR State OR trait ) AND ( MH "Adult+" OR adult* OR elder* OR aged OR geriatric* OR gerontol* OR College-student* OR university-student* OR men OR women OR man OR woman ) AND ( MH "Fibromyalgia" OR MH "Fatigue Syndrome, Chronic" OR Fibromyalgia* OR Muscular-Rheumatism* OR Fibrositis OR Fibrositides OR Diffuse-Myofascial-Pain-Syndrome* OR fibro OR Chronic-Fatigue OR Systemic-Exertion-Intolerance-Disease* OR Myalgic-Encephalomyelitis OR Postviral-Fatigue OR Chronic-Infectious-Mononucleosis-Like OR Royal-Free OR Musculoskeletal-Pain-Syndrome OR Myofibrositis OR Fibromyositis ) Search mode: Boolean/Phrase

*Embase*

('recreation'/de OR 'sport'/exp OR 'camping'/exp OR 'dancing'/exp OR 'exercise'/exp OR 'kinesiotherapy'/exp OR aerobic*:ab,ti OR anaerobic*:ab,ti OR exercise*:ab,ti OR plyometric*:ab,ti OR 'physical exertion*':ab,ti OR 'physical effort*':ab,ti OR 'physical activit*':ab,ti OR gymnastic*:ab,ti OR calisthenic*:ab,ti OR stretch*:ab,ti OR train*:ab,ti OR run:ab,ti OR runs:ab,ti OR running:ab,ti OR jog:ab,ti OR jogs:ab,ti OR jogging:ab,ti OR swim*:ab,ti OR walk*:ab,ti OR climb*:ab,ti OR 'weight lift*':ab,ti OR ambulat*:ab,ti OR fitness:ab,ti OR pilate*:ab,ti OR qigong:ab,ti OR 'qi gong':ab,ti OR danc*:ab,ti OR 'tai ji':ab,ti OR 'tai chi':ab,ti OR 'tai ji quan':ab,ti OR taiji:ab,ti OR taijiquan:ab,ti OR 'tai chi chuan':ab,ti OR yoga:ab,ti OR sport*:ab,ti OR athletic*:ab,ti OR baseball*:ab,ti OR softball*:ab,ti OR basketball*:ab,ti OR netball*:ab,ti OR bicycling:ab,ti OR cycling:ab,ti OR boxing*:ab,ti OR cricket:ab,ti OR football*:ab,ti OR rugb*:ab,ti OR golf*:ab,ti OR hockey*:ab,ti OR wrestl*:ab,ti OR 'martial art*':ab,ti OR 'hap di do':ab,ti OR judo:ab,ti OR karate:ab,ti OR jujitsu:ab,ti OR 'tae kwon do':ab,ti OR aikido:ab,ti OR wushu:ab,ti OR 'kung fu':ab,ti OR 'gong fu':ab,ti OR gongfu:ab,ti OR mountaineer*:ab,ti OR tennis:ab,ti OR racquetball:ab,ti OR racketball:ab,ti OR 'racket ball':ab,ti OR badminton:ab,ti OR lacrosse:ab,ti OR skating*:ab,ti OR skateboard*:ab,ti OR snowmobiling:ab,ti OR sledding:ab,ti OR skiing:ab,ti OR snowboard*:ab,ti OR soccer*:ab,ti OR volleyball*:ab,ti OR surfing:ab,ti OR rowing:ab,ti OR polo:ab,ti OR kayaking:ab,ti OR canoeing:ab,ti OR boating:ab,ti OR surfboarding:ab,ti OR recreation*:ab,ti OR garden*:ab,ti OR strength*:ab,ti OR resistance*:ab,ti) AND ('inflammation'/exp OR 'emotion'/exp OR 'microglia'/exp OR 'cytokine'/exp OR 'transforming growth factor'/exp OR 'astrocyte'/exp OR 'hla antigen'/exp OR 'cerebrospinal fluid'/exp OR 'c reactive protein'/exp OR 'brain derived neurotrophic factor'/exp OR 'glutamate transporter'/exp OR 'n acetylaspartic acid'/exp OR 'mental stress'/exp OR 'anxiety'/exp OR 'quality of life'/exp OR 'wellbeing'/exp OR neuroinflammat*:ab,ti OR inflammat*:ab,ti OR microglia*:ab,ti OR proinflammatory:ab,ti OR 'pro inflammatory':ab,ti OR cytokine*:ab,ti OR intercrine*:ab,ti OR chemokine*:ab,ti OR interleukin*:ab,ti OR 'il 6':ab,ti OR il6:ab,ti OR 'b-cell stimulatory-factor-2':ab,ti OR 'b cell differentiation factor 2':ab,ti OR 'bsf 2':ab,ti OR 'hybridoma growth factor*':ab,ti OR 'plasmacytoma growth factor*':ab,ti OR 'hepatocyte stimulating factor*':ab,ti OR 'mgi 2':ab,ti OR 'myeloid differentiation inducing protein*':ab,ti OR 'il 8':ab,ti OR il8:ab,ti OR 'monocyte derived neutrophil chemotactic factor*':ab,ti OR 'neutrophil activation factor*':ab,ti OR 'lymphocyte derived neutrophil activating peptide*':ab,ti OR 'monocyte derived neutrophil activating peptide*':ab,ti OR 'alveolar macrophage chemotactic factor i':ab,ti OR 'amcf i':ab,ti OR 'anionic neutrophil activating peptide*':ab,ti OR 'macrophage derived chemotactic factor*':ab,ti OR 'neutrophil chemotactic factor*':ab,ti OR 'granulocyte chemotactic peptide interleukin 8':ab,ti OR 'il 1':ab,ti OR 't helper factor*':ab,ti OR 'lymphocyte activating factor*':ab,ti OR 'macrophage cell factor*':ab,ti OR 'epidermal cell derived thymocyte activating factor*':ab,ti OR tnf:ab,ti OR 'tumor necrosis factor*':ab,ti OR ifn:ab,ti OR interferon*:ab,ti OR 'transforming growth factor*':ab,ti OR tgf:ab,ti OR microgliosis:ab,ti OR astrocyt*:ab,ti OR astrogli*:ab,ti OR hla:ab,ti OR 'human leukocyte antigen*':ab,ti OR 'leukocyte antigen*':ab,ti OR 'hl a':ab,ti OR 'major histocompatabilit*':ab,ti OR mhc:ab,ti OR 'mhc i':ab,ti OR 'immune response antigen*':ab,ti OR 'immune response associated antigen*':ab,ti OR 'i a antigen*':ab,ti OR 'ia like antigen*':ab,ti OR 'class ii mhc protein*':ab,ti OR 'class ii antigen*':ab,ti OR 'class ii histocompatibility antigen*':ab,ti OR 'ia antigen*':ab,ti OR 'immune associated antigen*':ab,ti OR 'mhc class i':ab,ti OR 'class i histocompatibility antigen*':ab,ti OR 'class i mhc':ab,ti OR 'class i antigen*':ab,ti OR 'mhc class ii':ab,ti OR 'il 4':ab,ti OR 'b cell growth factor 1':ab,ti OR 'b-cell-growth factor-i':ab,ti OR 'b cell proliferating factor*':ab,ti OR 'b cell stimulating factor 1':ab,ti OR 'bcgf 1':ab,ti OR binetrakin*:ab,ti OR 'bsf 1':ab,ti OR il4:ab,ti OR 'mast cell growth factor 2':ab,ti OR 'mcgf 2':ab,ti OR 'b cell stimulatory factor 1':ab,ti OR cxc:ab,ti OR 'c x c':ab,ti OR 'alpha chemokine*':ab,ti OR 'il 10':ab,ti OR il10:ab,ti OR 'csif 10':ab,ti OR 'cytokine synthesis inhibitory factor*':ab,ti OR 'il 12':ab,ti OR il12:ab,ti OR 'natural killer cell stimulatory factor*':ab,ti OR 'cytotoxic lymphocyte maturation factor*':ab,ti OR 'edodekin alfa':ab,ti OR 'il 11':ab,ti OR il11:ab,ti OR 'adipogenesis inhibitory factor*':ab,ti OR cachectin*:ab,ti OR 'gamma interferon*':ab,ti OR 'gm csfil 4':ab,ti OR 'cerebrospinal fluid*':ab,ti OR 'cerebro spinal fluid*':ab,ti OR cx3cl1:ab,ti OR neurotactin*:ab,ti OR fractalkine*:ab,ti OR crp:ab,ti OR 'c reactive protein*':ab,ti OR hscrp:ab,ti OR bdnf:ab,ti OR 'brain derived neurotrophic factor*':ab,ti OR 'glutamate specific neurotransmitter transporter*':ab,ti OR 'glutamate plasma membrane transporter protein*':ab,ti OR 'sodium glutamate cotransporter*':ab,ti OR 'sodium glutamate transporter*':ab,ti OR 'excitatory amino acid transporter protein*':ab,ti OR 'excitatory amino acid transport protein*':ab,ti OR 'eaat protein*':ab,ti OR 'myo inositol*':ab,ti OR 'n acetylaspartate':ab,ti OR 'n acetyl l aspartate':ab,ti OR 'n acetyl aspartate':ab,ti OR 'n acetylaspartic acid':ab,ti OR 'acetyl aspartic acid':ab,ti OR tnaa:ab,ti OR emotion*:ab,ti OR valence:ab,ti OR arousal:ab,ti OR anxiet*:ab,ti OR stress*:ab,ti OR affect:ab,ti OR feel*:ab,ti OR mood*:ab,ti OR angst:ab,ti OR nervous*:ab,ti OR 'anxious* or quality-of-life':ab,ti OR 'life quality':ab,ti OR 'well being':ab,ti OR wellness:ab,ti OR wellbeing:ab,ti OR state:ab,ti OR trait:ab,ti) AND ('adult'/exp OR adult*:ab,ti OR elder*:ab,ti OR aged:ab,ti OR geriatric*:ab,ti OR gerontol*:ab,ti OR 'college student*':ab,ti OR 'university student*':ab,ti OR men:ab,ti OR women:ab,ti OR man:ab,ti OR woman:ab,ti) AND ('fibromyalgia'/exp OR 'chronic fatigue syndrome'/de OR fibromyalgia*:ab,ti OR 'muscular rheumatism*':ab,ti OR fibrositis:ab,ti OR fibrositides:ab,ti OR 'diffuse myofascial pain syndrome*':ab,ti OR fibro:ab,ti OR 'chronic fatigue':ab,ti OR 'systemic exertion intolerance disease*':ab,ti OR 'myalgic encephalomyelitis':ab,ti OR 'postviral fatigue':ab,ti OR 'chronic infectious mononucleosis like':ab,ti OR 'royal free':ab,ti OR 'musculoskeletal pain syndrome':ab,ti OR myofibrositis:ab,ti OR fibromyositis:ab,ti) AND ('article'/it OR 'article in press'/it OR 'review'/it)

*CENTRAL*

Search Name: Taylor Fibromyalgia Exercise Summer 2023
Date Run: 20/07/2023 04:39:37
Comment:

ID Search Hits
#1 MeSH descriptor: [Fibromyalgia] explode all trees 1975
#2 MeSH descriptor: [Fatigue Syndrome, Chronic] explode all trees 498
#3 (Fibromyalgia* OR Muscular-Rheumatism* OR Fibrositis OR Fibrositides OR Diffuse-Myofascial-Pain-Syndrome* OR fibro OR Chronic-Fatigue OR Systemic-Exertion-Intolerance-Disease* OR Myalgic-Encephalomyelitis OR Postviral-Fatigue OR Chronic-Infectious-Mononucleosis-Like OR Royal-Free OR Musculoskeletal-Pain-Syndrome OR Myofibrositis OR Fibromyositis):ti,ab,kw 5569
#4 #1 OR #2 OR #3 5604
#5 MeSH descriptor: [Adult] explode all trees 585624
#6 (adult* OR elder* OR aged OR geriatric* OR gerontol* OR College-student* OR university-student* OR men OR women OR man OR woman):ti,ab,kw 1113034
#7 MeSH descriptor: [Inflammation] explode all trees 18242
#8 MeSH descriptor: [Microglia] explode all trees 42
#9 MeSH descriptor: [Cytokines] explode all trees 24286
#10 MeSH descriptor: [Transforming Growth Factors] explode all trees 36
#11 MeSH descriptor: [Astrocytes] explode all trees 22
#12 MeSH descriptor: [HLA Antigens] explode all trees 794
#13 MeSH descriptor: [Histocompatibility Antigens Class II] explode all trees 477
#14 MeSH descriptor: [Histocompatibility Antigens Class I] explode all trees 543
#15 MeSH descriptor: [Cerebrospinal Fluid] explode all trees 672
#16 MeSH descriptor: [C-Reactive Protein] explode all trees 5572
#17 MeSH descriptor: [Brain-Derived Neurotrophic Factor] explode all trees 472
#18 MeSH descriptor: [Glutamate Plasma Membrane Transport Proteins] explode all trees 6
#19 MeSH descriptor: [Emotions] explode all trees 39427
#20 MeSH descriptor: [Stress, Psychological] explode all trees 7949
#21 MeSH descriptor: [Anxiety] explode all trees 13136
#22 MeSH descriptor: [Quality of Life] explode all trees 43718
#23 (neuroinflammat* OR inflammat* OR Microglia* OR proinflammatory OR pro-inflammatory OR cytokine* OR Intercrine* OR chemokine* OR interleukin* OR IL-6 OR IL6 OR B-Cell Stimulatory-Factor-2 OR B-Cell-Differentiation-Factor-2 OR BSF-2 OR Hybridoma-Growth-Factor* OR Plasmacytoma-Growth-Factor* OR Hepatocyte-Stimulating-Factor* OR MGI-2 OR Myeloid-Differentiation-Inducing-Protein* OR IL-8 OR IL8 OR Monocyte-Derived-Neutrophil-Chemotactic-Factor* OR Neutrophil-Activation-Factor* OR Lymphocyte-Derived-Neutrophil-Activating-Peptide* OR Monocyte-Derived-Neutrophil-Activating-Peptide* OR Alveolar-Macrophage-Chemotactic-Factor-I OR AMCF-I OR Anionic-Neutrophil-Activating-Peptide* OR Macrophage-Derived-Chemotactic-Factor* OR Neutrophil-Chemotactic-Factor* OR Granulocyte-Chemotactic-Peptide-Interleukin-8 OR IL-1 OR T-Helper-Factor* OR Lymphocyte-Activating-Factor* OR Macrophage-Cell-Factor* OR Epidermal-Cell-Derived-Thymocyte-Activating-Factor* OR TNF OR Tumor-necrosis-factor* OR IFN OR Interferon* OR transforming-growth-factor* OR TGF OR microgliosis OR astrocyt* OR astrogli* OR HLA OR Human-leukocyte-antigen* OR Leukocyte-antigen* OR HL-A OR Major-histocompatabilit* OR MHC OR MHC-I OR Immune-response-antigen* OR Immune-response-associated-antigen* OR I-A-Antigen* OR Ia-Like-Antigen* OR Class-II-MHC-Protein* OR Class-II-Antigen* OR Class-II-Histocompatibility-Antigen* OR IA-Antigen* OR Immune-Associated-Antigen* OR MHC-Class-I OR Class-I-Antigen* OR Class-I-Histocompatibility-Antigen* OR Class-I-MHC OR Class-I-Antigen* OR MHC-class-II OR IL-4 OR B-Cell-Growth-Factor-1 OR B-Cell-Growth Factor-I OR B-Cell-Proliferating-Factor* OR B-Cell-Stimulating-Factor-1 OR B-Cell-Stimulatory-Factor-1 OR BCGF-1 OR Binetrakin* OR BSF-1 OR IL4 OR Mast-Cell-Growth-Factor-2 OR MCGF-2 OR B-Cell-Stimulatory-Factor-1 OR CXC OR C-X-C OR alpha-Chemokine* OR IL-10 OR IL10 OR CSIF-10 OR Cytokine-Synthesis-Inhibitory-Factor* OR IL-12 OR IL12 OR Natural-Killer-Cell-Stimulatory-Factor* OR Cytotoxic-Lymphocyte-Maturation-Factor* OR Edodekin-Alfa OR IL-11 OR IL11 OR Adipogenesis-Inhibitory-Factor* OR Cachectin* OR gamma-Interferon* OR GM-CSFIL-4 OR Cerebrospinal-fluid* OR Cerebro-Spinal-Fluid* OR CX3CL1 OR Neurotactin* OR Fractalkine* OR CRP OR c-reactive-protein* OR hsCRP OR BDNF OR Brain-derived-neurotrophic-factor* OR Glutamate-Specific-Neurotransmitter-Transporter* OR Glutamate-Plasma-Membrane-Transporter-Protein* OR Sodium-Glutamate-Cotransporter* OR Sodium-Glutamate-Transporter* OR Excitatory-Amino-Acid-Transporter-Protein* OR Excitatory-Amino-Acid-Transport-Protein* OR EAAT-Protein* OR myo-inositol* OR N-acetylaspartate OR N-acetyl-L-aspartate OR N-acetyl-aspartate OR N-acetylaspartic-acid OR Acetyl-aspartic-acid OR tNAA OR Emotion* OR Valence OR Arousal OR Anxiet* OR Stress* OR Affect OR Feel* OR Mood* OR Angst OR Nervous* OR Anxious* OR Quality-of-life OR Life-quality OR Well-being OR Wellness OR Wellbeing OR State OR trait):ti,ab,kw 456630
#24 #5 OR #6 1113034
#25 #7 OR #8 OR #9 OR #10 OR #11 OR #12 OR #13 OR #14 OR #15 OR #16 OR #17 OR #18 OR #19 OR #20 OR #21 OR #22 OR #23 500084
#26 MeSH descriptor: [Sports] explode all trees 20899
#27 MeSH descriptor: [Recreation] explode all trees 22812
#28 MeSH descriptor: [Exercise] explode all trees 38297
#29 MeSH descriptor: [Exercise Therapy] explode all trees 19609
#30 MeSH descriptor: [Physical Exertion] explode all trees 4247
#31 MeSH descriptor: [Exercise Movement Techniques] explode all trees 3207
#32 (Exercise* OR Plyometric* OR Physical-Exertion* OR physical-effort* OR physical-activit* OR Gymnastic* OR Calisthenic* OR stretch* OR train* OR run OR runs OR running OR jog OR jogs OR jogging OR swim* OR walk* OR climb* OR weight-lift* OR Ambulat* OR Fitness OR pilate* OR qigong OR qi-gong OR ch’i-kung OR danc* OR tai-ji OR tai-chi OR tai-ji-quan OR taiji OR taijiquan OR t’ai-chi OR tai-chi-chuan OR yoga OR Sport* OR athletic* OR baseball* OR softball* OR basketball* OR netball* OR bicycling OR cycling OR boxing* OR cricket OR football* OR rugb* OR golf* OR hockey* OR wrestl* OR martial-art* OR hap-di-do OR judo OR karate OR jujitsu OR tae-kwon-do OR aikido OR wushu OR kung-fu OR gong-fu OR gongfu OR mountaineer* OR tennis OR Racquetball OR Racketball OR Racket-Ball OR Badminton OR Lacrosse OR skating* OR skateboard* OR snowmobiling OR sledding OR skiing OR snowboard* OR soccer* OR volleyball* OR surfing OR rowing OR polo OR kayaking OR canoeing OR boating OR surfboarding OR recreation* OR garden* OR strength* OR resistance*):ti,ab,kw 382878
#33 #26 OR #27 OR #28 OR #29 OR #30 OR #31 OR #32 383675
#34 #4 AND #24 AND #25 AND #33 723

*Academic Search Premier*

( DE "SPORTS" OR DE "AERODYNAMICS in sports" OR DE "AERONAUTICAL sports" OR DE "AMATEUR sports" OR DE "AQUATIC sports" OR DE "BALL games" OR DE "BASEBALL" OR DE "COLLEGE sports" OR DE "COMBAT sports" OR DE "CONTACT sports" OR DE "CROSS-training (Sports)" OR DE "DISC golf" OR DE "EISSTOCKSCHIESSEN (Sports)" OR DE "ENDURANCE sports" OR DE "EXTREME sports" OR DE "FANTASY sports" OR DE "GAELIC games" OR DE "GOODWILL Games" OR DE "GYMNASTICS" OR DE "HOCKEY" OR DE "INDIVIDUAL sports" OR DE "KNIFE throwing" OR DE "LOG-chopping (Sports)" OR DE "MILITARY sports" OR DE "MOTORSPORTS" OR DE "OLYMPIC Games" OR DE "PARKOUR" OR DE "PROFESSIONAL sports" OR DE "RACKET games" OR DE "RECREATIONAL sports" OR DE "RODEOS" OR DE "ROLLER skating" OR DE "SCHOOL sports" OR DE "SENIOR Olympics" OR DE "SHOOTING (Sports)" OR DE "SKATEBOARDING" OR DE "SOFTBALL" OR DE "SPORTS for older people" OR DE "SPORTS for people with disabilities" OR DE "TEAM sports" OR DE "TEAMWORK (Sports)" OR DE "TOMAHAWK throwing" OR DE "TRACEURS" OR DE "WINTER sports" OR DE "WOMEN'S sports" OR DE "RECREATION" OR DE "FAMILY recreation" OR DE "GAMES" OR DE "HOBBIES" OR DE "OUTDOOR recreation" OR DE "PLAY" OR DE "RECREATIONAL sports" OR DE "RECREATIONAL therapy" OR DE "GARDENING" OR DE "EXERCISE" OR DE "ABDOMINAL exercises" OR DE "AEROBIC exercises" OR DE "ANAEROBIC exercises" OR DE "AQUATIC exercises" OR DE "ARM exercises" OR DE "BACK exercises" OR DE "BLOOD flow restriction training" OR DE "BREATHING exercises" OR DE "BREEMA" OR DE "BUTTOCKS exercises" OR DE "CALISTHENICS" OR DE "CHAIR exercises" OR DE "CHEST exercises" OR DE "CIRCUIT training" OR DE "COMPOUND exercises" OR DE "COOLDOWN" OR DE "CORRECTIVE exercise" OR DE "DO-in" OR DE "EXERCISE adherence" OR DE "EXERCISE for couples" OR DE "EXERCISE for men" OR DE "EXERCISE for middle-aged persons" OR DE "EXERCISE for older people" OR DE "EXERCISE for people with disabilities" OR DE "EXERCISE for women" OR DE "EXERCISE therapy" OR DE "EXERCISE video games" OR DE "FACIAL exercises" OR DE "FALUN gong exercises" OR DE "FOOT exercises" OR DE "GYMNASTICS" OR DE "HAND exercises" OR DE "HATHA yoga" OR DE "HIP exercises" OR DE "ISOKINETIC exercise" OR DE "ISOLATION exercises" OR DE "ISOMETRIC exercise" OR DE "ISOTONIC exercise" OR DE "KEGEL exercises" OR DE "KNEE exercises" OR DE "LEG exercises" OR DE "LIANGONG" OR DE "METABOLIC equivalent" OR DE "MULAN quan" OR DE "MUSCLE strength" OR DE "PILATES method" OR DE "PLYOMETRICS" OR DE "QI gong" OR DE "REDUCING exercises" OR DE "RUNNING" OR DE "SHOULDER exercises" OR DE "STRENGTH training" OR DE "TAI chi" OR DE "TREADMILL exercise" OR DE "WHEELCHAIR workouts" OR DE "YOGA" OR DE "PHYSICAL activity" OR DE "PHYSICAL fitness" OR DE "ANAEROBIC exercises" OR DE "ASTROLOGY & physical fitness" OR DE "BODYBUILDING" OR DE "CARDIOPULMONARY fitness" OR DE "CARDIOVASCULAR fitness" OR DE "CIRCUIT training" OR DE "COMPOUND exercises" OR DE "EXERCISE tolerance" OR DE "ISOLATION exercises" OR DE "LIANGONG" OR DE "MUSCLE strength" OR DE "PERIODIZATION training" OR DE "PHYSICAL fitness for men" OR DE "PHYSICAL fitness for middle-aged persons" OR DE "PHYSICAL fitness for older people" OR DE "PHYSICAL fitness for people with disabilities" OR DE "PHYSICAL fitness for women" OR DE "SPORTS participation" OR DE "EXERCISE therapy" OR DE "EXERCISE therapy for older people" OR DE "SWEDISH gymnastics" OR DE "MOVEMENT therapy" OR DE "DANCE therapy" OR DE "DANCE" OR ( Exercise* OR Plyometric* OR Physical-Exertion* OR physical-effort* OR physical-activit* OR Gymnastic* OR Calisthenic* OR stretch* OR train* OR run OR runs OR running OR jog OR jogs OR jogging OR swim* OR walk* OR climb* OR weight-lift* OR Ambulat* OR Fitness OR pilate* OR qigong OR qi-gong OR ch’i-kung OR danc* OR tai-ji OR tai-chi OR tai-ji-quan OR taiji OR taijiquan OR t’ai-chi OR tai-chi-chuan OR yoga OR Sport* OR athletic* OR baseball* OR softball* OR basketball* OR netball* OR bicycling OR cycling OR boxing* OR cricket OR football* OR rugb* OR golf* OR hockey* OR wrestl* OR martial-art* OR hap-di-do OR judo OR karate OR jujitsu OR tae-kwon-do OR aikido OR wushu OR kung-fu OR gong-fu OR gongfu OR mountaineer* OR tennis OR Racquetball OR Racketball OR Racket-Ball OR Badminton OR Lacrosse OR skating* OR skateboard* OR snowmobiling OR sledding OR skiing OR snowboard* OR soccer* OR volleyball* OR surfing OR rowing OR polo OR kayaking OR canoeing OR boating OR surfboarding OR recreation* OR garden* OR strength* OR resistance* ) ) AND ( DE "FIBROMYALGIA" OR DE "CHRONIC fatigue syndrome" OR ( Fibromyalgia* OR Muscular-Rheumatism* OR Fibrositis OR Fibrositides OR Diffuse-Myofascial-Pain-Syndrome* OR fibro OR Chronic-Fatigue OR Systemic-Exertion-Intolerance-Disease* OR Myalgic-Encephalomyelitis OR Postviral-Fatigue OR Chronic-Infectious-Mononucleosis-Like OR Royal-Free OR Musculoskeletal-Pain-Syndrome OR Myofibrositis OR Fibromyositis ) ) AND ( DE "ADULTS" OR DE "ADULT children" OR DE "MIDDLE age" OR DE "MIDDLE-aged persons" OR DE "OLD age" OR DE "OLDER people" OR DE "YOUNG adults" OR ( adult* OR elder* OR aged OR geriatric* OR gerontol* OR College-student* OR university-student* OR men OR women OR man OR woman ) ) AND ( DE "INFLAMMATION" OR DE "NEUROINFLAMMATION" OR DE "MICROGLIA" OR DE "CYTOKINES" OR DE "CHEMOKINES" OR DE "COLONY-stimulating factors (Physiology)" OR DE "FRACTALKINE" OR DE "GROWTH factors" OR DE "HEPATOCYTE growth factor" OR DE "INTERLEUKIN-12" OR DE "INTERLEUKIN-13" OR DE "INTERLEUKIN-18" OR DE "INTERLEUKIN-21" OR DE "LEUKEMIA inhibitory factor" OR DE "LEUKOLYSINS" OR DE "LYMPHOKINES" OR DE "MACROPHAGE activating factors" OR DE "MONOKINES" OR DE "PLEIOTROPHIN" OR DE "THYMIC stromal lymphopoietin" OR DE "TUMOR necrosis factors" OR DE "TRANSFORMING growth factors" OR DE "TRANSFORMING growth factors-beta" OR DE "ASTROCYTES" OR DE "HLA histocompatibility antigens" OR DE "HLA class II antigens" OR DE "HLA-B27 antigen" OR DE "HISTOCOMPATIBILITY class I antigens" OR DE "CEREBROSPINAL fluid" OR DE "BLOOD-brain barrier" OR DE "CEREBROSPINAL fluid proteins" OR DE "C-reactive protein" OR DE "BRAIN-derived neurotrophic factor" OR DE "NEUROTROPHINS" OR DE "EMOTIONS" OR DE "ABJECTION" OR DE "AFFECTIVE forecasting (Psychology)" OR DE "AGITATION (Psychology)" OR DE "AMBIVALENCE" OR DE "ANGER" OR DE "AUTONOMY (Psychology)" OR DE "AVERSION" OR DE "AWE" OR DE "BASHFULNESS" OR DE "BEREAVEMENT" OR DE "CALMNESS" OR DE "CATHARSIS" OR DE "CHEERFULNESS" OR DE "COMPASSION" OR DE "CONTROL (Psychology)" OR DE "COWARDICE" OR DE "CRYING" OR DE "DEFEAT (Psychology)" OR DE "DESIRE" OR DE "DESPAIR" OR DE "DISAPPOINTMENT" OR DE "DISCONTENT" OR DE "DISPLACEMENT (Psychology)" OR DE "ELATION" OR DE "EMBARRASSMENT" OR DE "EMOTION regulation" OR DE "EMOTIONAL conditioning" OR DE "EMOTIONAL shutdown (Psychology)" OR DE "EMOTIONAL state" OR DE "EMOTIONS & cognition" OR DE "EMPATHY" OR DE "ENTHUSIASM" OR DE "ENVY" OR DE "ESCAPE (Psychology)" OR DE "FACIAL expression & emotions (Psychology)" OR DE "FEAR" OR DE "FRAGILITY (Psychology)" OR DE "FRUSTRATION" OR DE "GRATITUDE" OR DE "GRIEF" OR DE "GUILT (Psychology)" OR DE "HAN (Psychology)" OR DE "HAPPINESS" OR DE "HATE" OR DE "HELPLESSNESS (Psychology)" OR DE "HOMESICKNESS" OR DE "HOPE" OR DE "HORROR" OR DE "HOSTILITY" OR DE "HUMILIATION" OR DE "IMPULSE (Psychology)" OR DE "INNOCENCE (Psychology)" OR DE "INTIMACY (Psychology)" OR DE "JEALOUSY" OR DE "JOY" OR DE "LANGUAGE & emotions" OR DE "LAUGHTER" OR DE "LOVE" OR DE "MELANCHOLY" OR DE "MOOD (Psychology)" OR DE "MUSIC & emotions" OR DE "NOSTALGIA" OR DE "PATHOS" OR DE "PERSONALITY & emotions" OR DE "PLEASANTNESS & unpleasantness (Psychology)" OR DE "PLEASURE" OR DE "PREJUDICES" OR DE "PSYCHOLOGICAL burnout" OR DE "PSYCHOLOGICAL distress" OR DE "PSYCHOLOGICAL stress" OR DE "REGRET" OR DE "REJECTION (Psychology)" OR DE "RELATEDNESS (Psychology)" OR DE "RELIEF (Psychology)" OR DE "REMORSE" OR DE "RESENTMENT" OR DE "RESIGNATION (Psychology)" OR DE "SADNESS" OR DE "SATISFACTION" OR DE "SELF-confidence" OR DE "SELF-consciousness (Sensitivity)" OR DE "SENTIMENTALISM" OR DE "SHAME" OR DE "SURPRISE" OR DE "SYMPATHY" OR DE "TEMPER" OR DE "TENDERNESS (Psychology)" OR DE "TIMIDITY" OR DE "TRANSFERENCE (Psychology)" OR DE "WONDER" OR DE "WORRY" OR DE "ACUTE stress disorder" OR DE "ANXIETY" OR DE "BURDEN of care" OR DE "CROWDING stress" OR DE "FINANCIAL stress" OR DE "HOLIDAY stress" OR DE "IMMOBILIZATION stress" OR DE "JOB stress" OR DE "MINORITY stress" OR DE "POST-traumatic stress" OR DE "POST-traumatic stress disorder" OR DE "PSYCHOLOGICAL burnout" OR DE "SECONDARY traumatic stress" OR DE "STRESS tolerance (Psychology)" OR DE "TIME pressure" OR DE "ANXIETY" OR DE "QUALITY of life" OR DE "RELATIONSHIP quality" OR DE "WELL-being" OR ( neuroinflammat* OR inflammat* OR Microglia* OR proinflammatory OR pro-inflammatory OR cytokine* OR Intercrine* OR chemokine* OR interleukin* OR IL-6 OR IL6 OR B-Cell Stimulatory-Factor-2 OR B-Cell-Differentiation-Factor-2 OR BSF-2 OR Hybridoma-Growth-Factor* OR Plasmacytoma-Growth-Factor* OR Hepatocyte-Stimulating-Factor* OR MGI-2 OR Myeloid-Differentiation-Inducing-Protein* OR IL-8 OR IL8 OR Monocyte-Derived-Neutrophil-Chemotactic-Factor* OR Neutrophil-Activation-Factor* OR Lymphocyte-Derived-Neutrophil-Activating-Peptide* OR Monocyte-Derived-Neutrophil-Activating-Peptide* OR Alveolar-Macrophage-Chemotactic-Factor-I OR AMCF-I OR Anionic-Neutrophil-Activating-Peptide* OR Macrophage-Derived-Chemotactic-Factor* OR Neutrophil-Chemotactic-Factor* OR Granulocyte-Chemotactic-Peptide-Interleukin-8 OR IL-1 OR T-Helper-Factor* OR Lymphocyte-Activating-Factor* OR Macrophage-Cell-Factor* OR Epidermal-Cell-Derived-Thymocyte-Activating-Factor* OR TNF OR Tumor-necrosis-factor* OR IFN OR Interferon* OR transforming-growth-factor* OR TGF OR microgliosis OR astrocyt* OR astrogli* OR HLA OR Human-leukocyte-antigen* OR Leukocyte-antigen* OR HL-A OR Major-histocompatabilit* OR MHC OR MHC-I OR Immune-response-antigen* OR Immune-response-associated-antigen* OR I-A-Antigen* OR Ia-Like-Antigen* OR Class-II-MHC-Protein* OR Class-II-Antigen* OR Class-II-Histocompatibility-Antigen* OR IA-Antigen* OR Immune-Associated-Antigen* OR MHC-Class-I OR Class-I-Antigen* OR Class-I-Histocompatibility-Antigen* OR Class-I-MHC OR Class-I-Antigen* OR MHC-class-II OR IL-4 OR B-Cell-Growth-Factor-1 OR B-Cell-Growth Factor-I OR B-Cell-Proliferating-Factor* OR B-Cell-Stimulating-Factor-1 OR B-Cell-Stimulatory-Factor-1 OR BCGF-1 OR Binetrakin* OR BSF-1 OR IL4 OR Mast-Cell-Growth-Factor-2 OR MCGF-2 OR B-Cell-Stimulatory-Factor-1 OR CXC OR C-X-C OR alpha-Chemokine* OR IL-10 OR IL10 OR CSIF-10 OR Cytokine-Synthesis-Inhibitory-Factor* OR IL-12 OR IL12 OR Natural-Killer-Cell-Stimulatory-Factor* OR Cytotoxic-Lymphocyte-Maturation-Factor* OR Edodekin-Alfa OR IL-11 OR IL11 OR Adipogenesis-Inhibitory-Factor* OR Cachectin* OR gamma-Interferon* OR GM-CSFIL-4 OR Cerebrospinal-fluid* OR Cerebro-Spinal-Fluid* OR CX3CL1 OR Neurotactin* OR Fractalkine* OR CRP OR c-reactive-protein* OR hsCRP OR BDNF OR Brain-derived-neurotrophic-factor* OR Glutamate-Specific-Neurotransmitter-Transporter* OR Glutamate-Plasma-Membrane-Transporter-Protein* OR Sodium-Glutamate-Cotransporter* OR Sodium-Glutamate-Transporter* OR Excitatory-Amino-Acid-Transporter-Protein* OR Excitatory-Amino-Acid-Transport-Protein* OR EAAT-Protein* OR myo-inositol* OR N-acetylaspartate OR N-acetyl-L-aspartate OR N-acetyl-aspartate OR N-acetylaspartic-acid OR Acetyl-aspartic-acid OR tNAA OR Emotion* OR Valence OR Arousal OR Anxiet* OR Stress* OR Affect OR Feel* OR Mood* OR Angst OR Nervous* OR Anxious* OR Quality-of-life OR Life-quality OR Well-being OR Wellness OR Wellbeing OR State OR trait ) )

*SPORTDiscus*

( DE "SPORTS" OR DE "AQUATIC sports" OR DE "BALL games" OR DE "BASEBALL" OR DE "COMBAT sports" OR DE "CONTACT sports" OR DE "CROSS-training (Sports)" OR DE "DISC golf" OR DE "ENDURANCE sports" OR DE "EXTREME sports" OR DE "FANTASY sports" OR DE "GAELIC games" OR DE "GOODWILL Games" OR DE "GYMNASTICS" OR DE "HOCKEY" OR DE "INDIVIDUAL sports" OR DE "KINEMATICS in sports" OR DE "KNIFE throwing" OR DE "LOG-chopping (Sports)" OR DE "MILITARY sports" OR DE "MINORITIES in sports" OR DE "OLYMPIC Games" OR DE "PROFESSIONAL sports" OR DE "RACKET games" OR DE "RECREATIONAL sports" OR DE "RODEOS" OR DE "ROLLER skating" OR DE "SENIOR Olympics" OR DE "SHOOTING (Sports)" OR DE "SKATEBOARDING" OR DE "SOFTBALL" OR DE "SPORTS for older people" OR DE "SPORTS for people with disabilities" OR DE "TEAM sports" OR DE "TOMAHAWK throwing" OR DE "WINTER sports" OR DE "WOMEN'S sports" OR DE "EXERCISE" OR DE "OUTDOOR life" OR DE "SPORTS participation" OR DE "ABDOMINAL exercises" OR DE "AEROBIC exercises" OR DE "ANAEROBIC exercises" OR DE "AQUATIC exercises" OR DE "ARM exercises" OR DE "BACK exercises" OR DE "BLOOD flow restriction training" OR DE "BREATHING exercises" OR DE "BREEMA" OR DE "BUTTOCKS exercises" OR DE "CALISTHENICS" OR DE "CHAIR exercises" OR DE "CHEST exercises" OR DE "CIRCUIT training" OR DE "COMPOUND exercises" OR DE "COOLDOWN" OR DE "DO-in" OR DE "EXERCISE adherence" OR DE "EXERCISE for couples" OR DE "EXERCISE for men" OR DE "EXERCISE for older people" OR DE "EXERCISE for people with disabilities" OR DE "EXERCISE for women" OR DE "EXERCISE therapy" OR DE "EXERCISE video games" OR DE "FACIAL exercises" OR DE "FALUN gong exercises" OR DE "FOOT exercises" OR DE "GYMNASTICS" OR DE "HAND exercises" OR DE "HATHA yoga" OR DE "HIP exercises" OR DE "ISOKINETIC exercise" OR DE "ISOLATION exercises" OR DE "ISOMETRIC exercise" OR DE "ISOTONIC exercise" OR DE "KEGEL exercises" OR DE "KNEE exercises" OR DE "LEG exercises" OR DE "LIANGONG" OR DE "MULAN quan" OR DE "PILATES method" OR DE "PLYOMETRICS" OR DE "QI gong" OR DE "REDUCING exercises" OR DE "RUNNING" OR DE "SHOULDER exercises" OR DE "STRENGTH training" OR DE "TAI chi" OR DE "TREADMILL exercise" OR DE "WHEELCHAIR workouts" OR DE "YOGA" OR DE "EXERCISE therapy" OR DE "EXERCISE therapy for older people" OR DE "SWEDISH gymnastics" OR DE "MOVEMENT therapy" OR DE "ANAEROBIC exercises" OR DE "BODYBUILDING" OR DE "PHYSICAL fitness for older people" OR DE "PHYSICAL activity" OR DE "PHYSICAL training & conditioning" OR ( Exercise* OR Plyometric* OR Physical-Exertion* OR physical-effort* OR physical-activit* OR Gymnastic* OR Calisthenic* OR stretch* OR train* OR run OR runs OR running OR jog OR jogs OR jogging OR swim* OR walk* OR climb* OR weight-lift* OR Ambulat* OR Fitness OR pilate* OR qigong OR qi-gong OR ch’i-kung OR danc* OR tai-ji OR tai-chi OR tai-ji-quan OR taiji OR taijiquan OR t’ai-chi OR tai-chi-chuan OR yoga OR Sport* OR athletic* OR baseball* OR softball* OR basketball* OR netball* OR bicycling OR cycling OR boxing* OR cricket OR football* OR rugb* OR golf* OR hockey* OR wrestl* OR martial-art* OR hap-di-do OR judo OR karate OR jujitsu OR tae-kwon-do OR aikido OR wushu OR kung-fu OR gong-fu OR gongfu OR mountaineer* OR tennis OR Racquetball OR Racketball OR Racket-Ball OR Badminton OR Lacrosse OR skating* OR skateboard* OR snowmobiling OR sledding OR skiing OR snowboard* OR soccer* OR volleyball* OR surfing OR rowing OR polo OR kayaking OR canoeing OR boating OR surfboarding OR recreation* OR garden* OR strength* OR resistance*)) AND ( DE "AFFECT (Psychology)" OR DE "INFLAMMATION" OR DE "NEUROINFLAMMATION” OR DE "EMOTIONS" OR DE "ABJECTION" OR DE "AGITATION (Psychology)" OR DE "AMBIVALENCE" OR DE "ANGER" OR DE "AUTONOMY (Psychology)" OR DE "AVERSION" OR DE "AWE" OR DE "BASHFULNESS" OR DE "BELIEF & doubt" OR DE "BEREAVEMENT" OR DE "CALMNESS" OR DE "CATHARSIS" OR DE "CHEERFULNESS" OR DE "COMPASSION" OR DE "CONTROL (Psychology)" OR DE "COWARDICE" OR DE "CRYING" OR DE "DEFEAT (Psychology)" OR DE "DESIRE" OR DE "DESPAIR" OR DE "DISAPPOINTMENT" OR DE "DISCONTENT" OR DE "DISPLACEMENT (Psychology)" OR DE "ELATION" OR DE "EMBARRASSMENT" OR DE "EMOTION regulation" OR DE "EMOTIONAL conditioning" OR DE "EMOTIONAL shutdown (Psychology)" OR DE "EMOTIONAL state" OR DE "EMOTIONS & cognition" OR DE "EMPATHY" OR DE "ENTHUSIASM" OR DE "ENVY" OR DE "ESCAPE (Psychology)" OR DE "FACIAL expression & emotions (Psychology)" OR DE "FEAR" OR DE "FRAGILITY (Psychology)" OR DE "FRUSTRATION" OR DE "GRATITUDE" OR DE "GRIEF" OR DE "GUILT (Psychology)" OR DE "HAN (Psychology)" OR DE "HAPPINESS" OR DE "HATE" OR DE "HELPLESSNESS (Psychology)" OR DE "HOMESICKNESS" OR DE "HOPE" OR DE "HORROR" OR DE "HOSTILITY" OR DE "HUMILIATION" OR DE "IMPULSE (Psychology)" OR DE "INNOCENCE (Psychology)" OR DE "INTIMACY (Psychology)" OR DE "JEALOUSY" OR DE "JOY" OR DE "LANGUAGE & emotions" OR DE "LAUGHTER" OR DE "LOVE" OR DE "MELANCHOLY" OR DE "MOOD (Psychology)" OR DE "MUSIC & emotions" OR DE "NOSTALGIA" OR DE "PATHOS" OR DE "PLEASANTNESS & unpleasantness (Psychology)" OR DE "PLEASURE" OR DE "PREJUDICES" OR DE "PSYCHOLOGICAL burnout" OR DE "PSYCHOLOGICAL distress" OR DE "PSYCHOLOGICAL stress" OR DE "REGRET" OR DE "REJECTION (Psychology)" OR DE "RELATEDNESS (Psychology)" OR DE "RELIEF (Psychology)" OR DE "REMORSE" OR DE "RESENTMENT" OR DE "RESIGNATION (Psychology)" OR DE "SADNESS" OR DE "SATISFACTION" OR DE "SELF-confidence" OR DE "SELF-consciousness (Sensitivity)" OR DE "SENTIMENTALISM" OR DE "SHAME" OR DE "SMILING" OR DE "SURPRISE" OR DE "SYMPATHY" OR DE "TEMPER" OR DE "TEMPER tantrums" OR DE "TENDERNESS (Psychology)" OR DE "TIMIDITY" OR DE "TRANSFERENCE (Psychology)" OR DE "WONDER" OR DE "WORRY" OR DE "CYTOKINES" OR DE "GROWTH factors" OR DE "ANXIETY" OR DE "COMPETITIVE state anxiety" OR DE "PERFORMANCE anxiety" OR DE "QUALITY of life" OR DE "HEALTH status indicators" OR DE "LIFESTYLES" OR DE "WELL-being" OR ( neuroinflammat* OR inflammat* OR Microglia* OR proinflammatory OR pro-inflammatory OR cytokine* OR Intercrine* OR chemokine* OR interleukin* OR IL-6 OR IL6 OR B-Cell Stimulatory-Factor-2 OR B-Cell-Differentiation-Factor-2 OR BSF-2 OR Hybridoma-Growth-Factor* OR Plasmacytoma-Growth-Factor* OR Hepatocyte-Stimulating-Factor* OR MGI-2 OR Myeloid-Differentiation-Inducing-Protein* OR IL-8 OR IL8 OR Monocyte-Derived-Neutrophil-Chemotactic-Factor* OR Neutrophil-Activation-Factor* OR Lymphocyte-Derived-Neutrophil-Activating-Peptide* OR Monocyte-Derived-Neutrophil-Activating-Peptide* OR Alveolar-Macrophage-Chemotactic-Factor-I OR AMCF-I OR Anionic-Neutrophil-Activating-Peptide* OR Macrophage-Derived-Chemotactic-Factor* OR Neutrophil-Chemotactic-Factor* OR Granulocyte-Chemotactic-Peptide-Interleukin-8 OR IL-1 OR T-Helper-Factor* OR Lymphocyte-Activating-Factor* OR Macrophage-Cell-Factor* OR Epidermal-Cell-Derived-Thymocyte-Activating-Factor* OR TNF OR Tumor-necrosis-factor* OR IFN OR Interferon* OR transforming-growth-factor* OR TGF OR microgliosis OR astrocyt* OR astrogli* OR HLA OR Human-leukocyte-antigen* OR Leukocyte-antigen* OR HL-A OR Major-histocompatabilit* OR MHC OR MHC-I OR Immune-response-antigen* OR Immune-response-associated-antigen* OR I-A-Antigen* OR Ia-Like-Antigen* OR Class-II-MHC-Protein* OR Class-II-Antigen* OR Class-II-Histocompatibility-Antigen* OR IA-Antigen* OR Immune-Associated-Antigen* OR MHC-Class-I OR Class-I-Antigen* OR Class-I-Histocompatibility-Antigen* OR Class-I-MHC OR Class-I-Antigen* OR MHC-class-II OR IL-4 OR B-Cell-Growth-Factor-1 OR B-Cell-Growth Factor-I OR B-Cell-Proliferating-Factor* OR B-Cell-Stimulating-Factor-1 OR B-Cell-Stimulatory-Factor-1 OR BCGF-1 OR Binetrakin* OR BSF-1 OR IL4 OR Mast-Cell-Growth-Factor-2 OR MCGF-2 OR B-Cell-Stimulatory-Factor-1 OR CXC OR C-X-C OR alpha-Chemokine* OR IL-10 OR IL10 OR CSIF-10 OR Cytokine-Synthesis-Inhibitory-Factor* OR IL-12 OR IL12 OR Natural-Killer-Cell-Stimulatory-Factor* OR Cytotoxic-Lymphocyte-Maturation-Factor* OR Edodekin-Alfa OR IL-11 OR IL11 OR Adipogenesis-Inhibitory-Factor* OR Cachectin* OR gamma-Interferon* OR GM-CSFIL-4 OR Cerebrospinal-fluid* OR Cerebro-Spinal-Fluid* OR CX3CL1 OR Neurotactin* OR Fractalkine* OR CRP OR c-reactive-protein* OR hsCRP OR BDNF OR Brain-derived-neurotrophic-factor* OR Glutamate-Specific-Neurotransmitter-Transporter* OR Glutamate-Plasma-Membrane-Transporter-Protein* OR Sodium-Glutamate-Cotransporter* OR Sodium-Glutamate-Transporter* OR Excitatory-Amino-Acid-Transporter-Protein* OR Excitatory-Amino-Acid-Transport-Protein* OR EAAT-Protein* OR myo-inositol* OR N-acetylaspartate OR N-acetyl-L-aspartate OR N-acetyl-aspartate OR N-acetylaspartic-acid OR Acetyl-aspartic-acid OR tNAA OR Emotion* OR Valence OR Arousal OR Anxiet* OR Stress* OR Affect OR Feel* OR Mood* OR Angst OR Nervous* OR Anxious* OR Quality-of-life OR Life-quality OR Well-being OR Wellness OR Wellbeing OR State OR trait ) ) AND ( ( DE "OLDER people" OR DE "GERIATRICS" OR DE "YOUNG adults" ) OR ( adult* OR elder* OR aged OR geriatric* OR gerontol* OR College-student* OR university-student* OR men OR women OR man OR woman ) ) AND ( ( DE "FIBROMYALGIA" OR DE "CHRONIC fatigue syndrome" ) OR ( Fibromyalgia* OR Muscular-Rheumatism* OR Fibrositis OR Fibrositides OR Diffuse-Myofascial-Pain-Syndrome* OR fibro OR Chronic-Fatigue OR Systemic-Exertion-Intolerance-Disease* OR Myalgic-Encephalomyelitis OR Postviral-Fatigue OR Chronic-Infectious-Mononucleosis-Like OR Royal-Free OR Musculoskeletal-Pain-Syndrome OR Myofibrositis OR Fibromyositis ) )

*PsycINFO*

(MAINSUBJECT.EXACT("Physical Fitness") OR MAINSUBJECT.EXACT.EXPLODE("Exercise") OR MAINSUBJECT.EXACT.EXPLODE("Physical Activity") OR MAINSUBJECT.EXACT("Physical Activity") OR MAINSUBJECT.EXACT.EXPLODE("Camping") OR MAINSUBJECT.EXACT.EXPLODE("Sports") OR MAINSUBJECT.EXACT.EXPLODE("Dance") OR tiab(aerobic* OR anaerobic* OR Exercise* OR Plyometric* OR Physical-Exertion* OR physical-effort* OR physical-activit* OR Gymnastic* OR Calisthenic* OR stretch* OR train* OR run OR runs OR running OR jog OR jogs OR jogging OR swim* OR walk* OR climb* OR weight-lift* OR Ambulat* OR Fitness OR pilate* OR qigong OR qi-gong OR danc* OR tai-ji OR tai-chi OR tai-ji-quan OR taiji OR taijiquan OR “tai chi chuan” OR yoga OR Sport* OR athletic* OR baseball* OR softball* OR basketball* OR netball* OR bicycling OR cycling OR boxing* OR cricket OR football* OR rugb* OR golf* OR hockey* OR wrestl* OR martial-art* OR “hap di do” OR judo OR karate OR jujitsu OR “tae kwon do” OR aikido OR wushu OR “kung fu” OR “gong fu” OR gongfu OR mountaineer* OR tennis OR Racquetball OR Racketball OR Racket-Ball OR Badminton OR Lacrosse OR skating* OR skateboard* OR snowmobiling OR sledding OR skiing OR snowboard* OR soccer* OR volleyball* OR surfing OR rowing OR polo OR kayaking OR canoeing OR boating OR surfboarding OR recreation* OR garden* OR strength* OR resistance* )) AND (MAINSUBJECT.EXACT.EXPLODE("Emotions") OR MAINSUBJECT.EXACT.EXPLODE("Inflammation") OR MAINSUBJECT.EXACT("Microglia") OR MAINSUBJECT.EXACT.EXPLODE("Cytokines") OR MAINSUBJECT.EXACT("Astrocytes") OR MAINSUBJECT.EXACT.EXPLODE("Inflammatory Markers") OR MAINSUBJECT.EXACT("Brain Derived Neurotrophic Factor") OR MAINSUBJECT.EXACT.EXPLODE("Stress") OR MAINSUBJECT.EXACT.EXPLODE("Quality of Life") OR MAINSUBJECT.EXACT.EXPLODE("Well Being") OR tiab(neuroinflammat* OR inflammat* OR Microglia* OR proinflammatory OR pro-inflammatory OR cytokine* OR Intercrine* OR chemokine* OR interleukin* OR IL-6 OR IL6 OR B-Cell Stimulatory-Factor-2 OR B-Cell-Differentiation-Factor-2 OR BSF-2 OR Hybridoma-Growth-Factor* OR Plasmacytoma-Growth-Factor* OR Hepatocyte-Stimulating-Factor* OR MGI-2 OR Myeloid-Differentiation-Inducing-Protein* OR IL-8 OR IL8 OR Monocyte-Derived-Neutrophil-Chemotactic-Factor* OR Neutrophil-Activation-Factor* OR Lymphocyte-Derived-Neutrophil-Activating-Peptide* OR Monocyte-Derived-Neutrophil-Activating-Peptide* OR Alveolar-Macrophage-Chemotactic-Factor-I OR AMCF-I OR Anionic-Neutrophil-Activating-Peptide* OR Macrophage-Derived-Chemotactic-Factor* OR Neutrophil-Chemotactic-Factor* OR Granulocyte-Chemotactic-Peptide-Interleukin-8 OR IL-1 OR T-Helper-Factor* OR Lymphocyte-Activating-Factor* OR Macrophage-Cell-Factor* OR Epidermal-Cell-Derived-Thymocyte-Activating-Factor* OR TNF OR Tumor-necrosis-factor* OR IFN OR Interferon* OR transforming-growth-factor* OR TGF OR microgliosis OR astrocyt* OR astrogli* OR HLA OR Human-leukocyte-antigen* OR Leukocyte-antigen* OR HL-A OR Major-histocompatabilit* OR MHC OR MHC-I OR Immune-response-antigen* OR Immune-response-associated-antigen* OR I-A-Antigen* OR Ia-Like-Antigen* OR Class-II-MHC-Protein* OR Class-II-Antigen* OR Class-II-Histocompatibility-Antigen* OR IA-Antigen* OR Immune-Associated-Antigen* OR MHC-Class-I OR Class-I-Antigen* OR Class-I-Histocompatibility-Antigen* OR Class-I-MHC OR Class-I-Antigen* OR MHC-class-II OR IL-4 OR B-Cell-Growth-Factor-1 OR B-Cell-Growth Factor-I OR B-Cell-Proliferating-Factor* OR B-Cell-Stimulating-Factor-1 OR B-Cell-Stimulatory-Factor-1 OR BCGF-1 OR Binetrakin* OR BSF-1 OR IL4 OR Mast-Cell-Growth-Factor-2 OR MCGF-2 OR B-Cell-Stimulatory-Factor-1 OR CXC OR C-X-C OR alpha-Chemokine* OR IL-10 OR IL10 OR CSIF-10 OR Cytokine-Synthesis-Inhibitory-Factor* OR IL-12 OR IL12 OR Natural-Killer-Cell-Stimulatory-Factor* OR Cytotoxic-Lymphocyte-Maturation-Factor* OR Edodekin-Alfa OR IL-11 OR IL11 OR Adipogenesis-Inhibitory-Factor* OR Cachectin* OR gamma-Interferon* OR GM-CSFIL-4 OR Cerebrospinal-fluid* OR Cerebro-Spinal-Fluid* OR CX3CL1 OR Neurotactin* OR Fractalkine* OR CRP OR c-reactive-protein* OR hsCRP OR BDNF OR Brain-derived-neurotrophic-factor* OR Glutamate-Specific-Neurotransmitter-Transporter* OR Glutamate-Plasma-Membrane-Transporter-Protein* OR Sodium-Glutamate-Cotransporter* OR Sodium-Glutamate-Transporter* OR Excitatory-Amino-Acid-Transporter-Protein* OR Excitatory-Amino-Acid-Transport-Protein* OR EAAT-Protein* OR myo-inositol* OR N-acetylaspartate OR N-acetyl-L-aspartate OR N-acetyl-aspartate OR N-acetylaspartic-acid OR Acetyl-aspartic-acid OR tNAA OR Emotion* OR Valence OR Arousal OR Anxiet* OR Stress* OR Affect OR Feel* OR Mood* OR Angst OR Nervous* OR Anxious* OR Quality-of-life OR Life-quality OR Well-being OR Wellness OR Wellbeing OR State OR trait)) AND (MAINSUBJECT.EXACT.EXPLODE("Fibromyalgia") OR MAINSUBJECT.EXACT.EXPLODE("Chronic Fatigue Syndrome") OR tiab(Fibromyalgia* OR Muscular-Rheumatism* OR Fibrositis OR Fibrositides OR Diffuse-Myofascial-Pain-Syndrome* OR fibro OR Chronic-Fatigue OR Systemic-Exertion-Intolerance-Disease* OR Myalgic-Encephalomyelitis OR Postviral-Fatigue OR Chronic-Infectious-Mononucleosis-Like OR Royal-Free OR Musculoskeletal-Pain-Syndrome OR Myofibrositis OR Fibromyositis) )
